# Compositional Variability of Essential Oils and Their Bioactivity in Native and Invasive *Erigeron* Species

**DOI:** 10.3390/molecules30142989

**Published:** 2025-07-16

**Authors:** Asta Judžentienė

**Affiliations:** 1 Institute of Biosciences, Life Sciences Center, Vilnius University, Saulėtekio Avenue 7, LT-10257 Vilnius, Lithuania; asta.judzentiene@gmc.vu.lt or asta.judzentiene@ftmc.lt; 2 Department of Organic Chemistry, Center for Physical Sciences and Technology, Saulėtekio Avenue 3, LT-10257 Vilnius, Lithuania

**Keywords:** *Erigeron*, f. *Asteraceae*, essential oils, limonene, *β*-ocimene matricaria ester, lachnophyllum ester, *β*-farnesene, *α*-bergamotene, modhephen-8-*β*-ol, dendrolasin, ledene oxide, antimicrobial activity, anticancer properties, insecticidal, larvicidal and repellent activities, allelopathic effects

## Abstract

To date, various species of *Erigeron* genus have been used both in the ethnopharmacology of numerous nations across the world and in contemporary herbal practices. The objective of this study is to revise the phytochemical data on the essential oils (EOs) of various fleabanes species and to evaluate the variability of their biological activities. Up to June 2025, this review provides an updated overview of 105 literature sources (published during last 25 years) related to 14 *Erigeron* sp. (native, naturalized, or invasive) which have been investigated extensively and are of the greatest significance. It summarizes the compositional variability of the EOs and their pharmacological and toxic effects, such as anti-inflammatory, anticancer, antiproliferative, skin regeneration, antioxidant, antifungal, antibacterial, insecticidal, larvicidal, repellent, and allelopathic activity. The EOs of each *Erigeron* species were characterized, and a chemical structure of 43 major constituents is presented herein. The most characteristic and prevalent compounds were found to be limonene, *δ*-3-carene, matricaria ester, lachnophyllum ester, germacrene D, *β*-caryophyllene, *β*-farnesene, *α*-bergamotene, *allo-aromadendrene*, etc., in the EOs from the *E. acris*, *E. annuus*, *E. bonariensis*, *E. canadensis*, *E. floribundus E. mucronatus*, and *E. speciosus* plants. Major constituents, such as borneol, bornyl acetate, modhephen-8-*β*-ol, *cis*-arteannuic alcohol, *β*-caryophyllene, and *τ*-cadinol, were found in the oils of *E. graveolens* (*Inula graveolens*). A paucity of data concerning *E. incanus* EOs was revealed, with the prevalence of 3-hydroxy-4-methoxy cinammic acid and thymol acetate noted in the oils. The EOs from *E. multiradiatus* and *E. sublyratus* were comprised mainly of matricaria and lachnophyllum esters. The available data on EOs of *E. ramosus* is limited, but the main constituents are known to be *α*-humulene, 1,8-cineole, eugenol, and globulol. The EOs containing appreciable amounts of matricaria and lachnophyllum esters exhibited strong anticancer, anti-inflammatory, antimicrobial, larvicidal, and repellent activities. Repellence is also related to borneol, bornyl acetate, caryophyllene derivatives, *τ*-cadinol, modhephen-8-*β*-ol, and *cis*-arteannuic alcohol. Cytotoxicity was determined due to the presence of limonene, *δ*-3-carene, *α*- and *β*-farnesene, (*E*)-*β*-ocimene, ledene oxide, sesquiphellandrene, and dendrolasin in the fleabanes EOs. Skin regeneration and antifungal properties were related to germacrene D; and anti-inflammatory effects were determined due to high amounts of limonene (*E*)-*β*-ocimene, lachnophyllum ester, and germacrene D. The antimicrobial properties of the oils were conditioned by appreciable quantities of limonene, *β*-pinene, 1,8-cineole, carvacrol, thymol acetae, *β*-eudesmol, 2,6,7,7*α*-tetrahydro-1,5-dimethyl-1H-indene-3-carboxaldehyde, caryophyllene and its oxide, *allo*-aromadendrene, *α*-humulene, farnesene, carvacrol, and eugenol. This review provides a foundation for further studies on volatile secondary metabolites to explore the potential sources of new biologically active compounds in *Erigeron* sp.

## 1. Introduction

The genus name *Erigeron* (first published in 1753) (*Asteraceae*) was accepted by World Flora Online (WFO, Plant List), and a number of accepted species names in the genus is approximately 450 [1]. Additionally, up to 480 species of plants in the genus *Erigeron* are listed on Plants of the World Online (POWO) as of June 2025 [2]. The genus comprises over 200 annual, biennial, and perennial species native to North and Central America, as well as 390 species distributed worldwide [3]. Plants of the genus have been classified within the tribe Astereae (f. *Asteraceae*), exhibiting a close relationship with both asters and the true daisies (bellis). It is important to note that fleabanes have been frequently misidentified as a species of asters, particularly within alpine environments. The taxonomic classification of plants into this genus has been a complicated process that has undergone modification over time.

The current global distribution, preferred climatic conditions, biological information, and morphological features of *Erigenon* have been well documented [1,2,3,4,5]. The plants (subshrubs, shrubs, or trees) are distinguished by its well-branched structure, with erect stems (glabrous or hairy) that bear plenty of white, lavender, or pink ray flowers, complemented by yellow disk flowers [4].

*Erigeron* has a long history of traditional uses in various ethno-cultures, and occupies an important place in traditional medicines all over the world. In the folk medicine of European countries, the roots of *E. acris* are applied to alleviate a range of ailments, including toothaches, arthritic pains, bruises, digestive disorders, and enteritis [6,7]. In Traditional Chinese Medicine, the plants of *E. acris* and *E. annuus* are widely used in various formulations to treat indigestion, enteritis, epidemic hepatitis, diabetes, hematuria, malaria, and obesity [8,9]. *E. bonariensis* is a well-known therapeutic herb in many cultures. Its properties have been utilized in ethnomedical practices for a variety of ailments, including cancer, diabetes, the management of age-related changes, antimicrobial properties, wound healing, diuretic effects, and the treatment of diarrhea and hemorrhoids [10,11,12,13]. Formulations of *E. breviscapus* are effective ethnopharmacological remedies in many Asian countries for a wide range of diseases, including cardiovascular, gastrointestinal, respiratory, and metabolic diseases [14,15]. The plants of *E. canadensis* are used for the treatment of acute toothache, otitis media, conjunctivitis, stomatitis, allergic diarrhea, wounds, swellings, and pain caused by arthritis in Chinese folk medicine [16,17]. The utilization of Canadian fleabanes in the folk medicine in the Northern regions of Pakistan is a long-standing practice [18,19]. The plant is employed in the treatment of a wide range of ailments, including pain, inflammation, fever, and, most notably, microbial infections such as urinary tract infections, respiratory tract infections, diarrhea, and dysentery [18]. In African folk medicine, *E. canadensis* was used to treat granuloma annulare, ringworm, eczema, sore throats, and urinary tract infections, as well as in medicinal baths [18,19]. *E. floribundus*, in addition to its established antimicrobial, analgesic, and anti-inflammatory properties, is a traditional remedy utilized in the treatment of malaria, a globally significant tropical parasitic disease, within the folk medicinal traditions of Cameroon and Uganda [20,21]. Moreover, the plant has multiple traditional uses in African countries, including healing stomach disorders, rheumatism, gout, cystitis, nephritis, dysmenorrhea, dental pains, skin problems, and headaches [21,22]. *E. graveolens* is another species that has been employed in both folk and modern medicine due to its antifungal, antibacterial, anti-inflammatory, and sedative properties [23]. *E. incana* plants are traditionally used as a tonic herb and for treatment of wounds in Yemen [24]. In India, the tribes of the Nilgiri Hills use *E. mucronatus* as a folk remedy for the treatment of a range of ailments, including diarrhea, dysentery, epilepsy, hemorrhages, paralysis, and diabetes, among others [25]. Preparation of *E. multiradiatus* plants are used by traditional healers and native people to cure hepatitis, meningitis, hemiparalysis, enteritis, diarrhea, rheumatism, and polyneuritis in Traditional Tibetan Medicine [26].

A considerable number of *Erigeron* species (native, casual, naturalized, or invasive) are employed as remedies in the ethnopharmacology of numerous countries; and the pivotal role of the essential oils (EOs) must be emphasized. EOs are defined as mixtures of fragrant compounds or as mixtures of fragrant and odorless substances [27]. Usually, EOs are aromatic volatile liquids, obtained mainly using steam distillation (hydro-distillation) of volatile organic compounds from plant material, which are produced by glandular trichomes and other secretory organs in different parts of plants. These secondary metabolites have been shown to play a multipurpose role in plant physiology, contributing to processes such as growth, defense, communication, reproduction, etc. However, volatile organic compounds have been found to have a significant impact on the bioactivity of herbal material.

The aim of the present study is to revise and summarize phytochemical data on the EOs of various *Erigeron* species that have been published during the last twenty-five years; to make comparisons between them; and to evaluate the variability in their biological activities. The objective of this review is to stimulate further studies in order to establish the molecular pathways in the observed activities and to explore potential sources of new biologically active compounds of the *Erigeron* genus.

## 2. Methodology

This review comprises data published between the years 2000 and 2025 concerning the chemical composition of EOs and their bioactivity of the *Erigeron* species which were predominantly investigated and are of the greatest significance, such as *E. acris*, *E. annuus*, *E. bonariensis*, *E. breviscapus*, *E. canadensis*, *E. floribundus*, *E. graveolens*, *E. incanus*, *E. mucronatus*, *E. multiradiatus*, *E. philadelphicus*, *E. strigosus*, *E. speciosus*, and *E. sublyratus*. This study examines compositional variability on the basis of major compounds, representing >5% of the total EO content. Furthermore, the origin of the Erigeron plant specimens is introduced, along with details concerning the oil preparation process.

Relevant information was gathered and synthesized using an online search engine of scientific databases such as Google Scholar (https://scholar.google.com (accessed on 5 May 2025)), PubMed (https://pubmed.ncbi.nlm.nih.gov (accessed on 5 May 2025)), Scopus (https://www.scopus.com/home.uri (accessed on 5 May 2025)), ScienceDirect (https://www.sciencedirect.com), Wiley Online Library (https://onlinelibrary.wiley.com/advanced/search (accessed on 5 May 2025)), and ACS Publications (https://pubs.acs.org (accessed on 5 May 2025)). The keywords, such as “*Erigeron* essential oil”, “*Conyza canadensis* essential oil”, “*Conyza graveolens* essential oil”, “*Erigeron multiradiatus* essential oil”, etc., were applied for this purpose.

A Principal Component Analysis (PCA) of major components and various biological activities of *E. bonariensis* and *E. canadensis* EOs was performed using Microsoft Excel Add-in Analyse-it 5.30 Standard Edition (Analyse-it Software, Ltd., Leeds, UK) (Supplementary Material, Figures S1 and S2).

## 3. Results

### 3.1. Erigeron acris L.

*Erigeron acris* (L.) (common names blue fleabane, bitter fleabane) is an annual, biennial, or rarely perennial plant, 15–50 cm in height. It is characterized by the following features: a taproot and a woody rhizome, a stem ascending, covered with bristles, oblong hairy leaves, and flowers, which are typically blue or blue-purple in color [28]. The plant is known by various other names, the most popular being *Aster erigeron* or *Erigeron alpinus* Lam. (illeg.) [1]. The species is native to Canada, northern parts of the United States, Asia, and most of Europe [2].

#### 3.1.1. Compositional Data of Essential Oils (EOs) from *Erigeron acris* L.

As demonstrated in Table 1, the available data concerning *E. acris* EOs is extremely limited during last quarter of a century [28,29]. Plant material was collected in Poland, where *E. acris* is a native taxon [30]. The optimal growing locations for this species are dry grasslands, fields, wastelands, and roadsides, where it can thrive in full-sun conditions [28,29].

**Figure 1:**
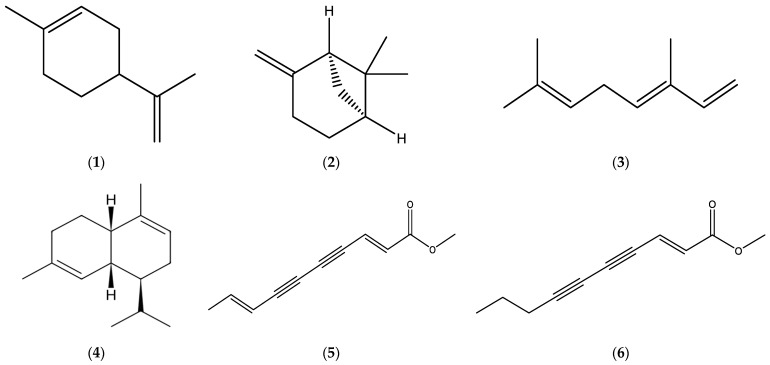
Chemical structures of limonene (**1**), *β*-pinene (**2**), *β*-ocimene (**3**), *α*-murolene (**4**), matricaria ester (**5**) (https://pubchem.ncbi.nlm.nih.gov/compound/Matricaria-ester (accessed on 6 May 2025)), and lachnophyllum ester (**6**) (https://pubchem.ncbi.nlm.nih.gov/compound/Lachnophyllum-ester (accessed on 6 May 2025)). Chemical structures of compounds were drawn using the ChemDraw (version 16.0) molecule editor.

Major constituents in the EOs derived from the herbal material of *E. acris* were determined to be some monoterpenes, namely limonene (**1**), *β*-pinene (**2**), and *β*-ocimene (**3**), and a sesquiterpene *α*-murolene (**4**), while the root oils were found to be rich in matricaria ester (**5**) and lachnophyllum ester (**6**) (Figure 1).

#### 3.1.2. Bioactivity (Anticancer and Antifungal Properties) of *E. acris* EOs

The antiproliferative and antifungal activities of the EOs from Polish *E. acris* roots and herbs were investigated by Nazaruk et al. [6]. A cell viability assay was performed in cultured fibroblasts, cancer cell lines (MCF-7 and MDA-MBA-231), endometrial adenocarcinoma (Ishikawa), and colon adenocarcinoma (DLD-1) cells. The root oils demonstrated the highest antiproliferative activity in the MCF-7 cells (IC_50_ = 14.5 μg/mL) and could be applied for breast cancer treatment [6,31]. Furthermore, the remarkable antifungal properties of the bitter fleabane EOs were estimated against various strains of five *Candida* species (*C. albicans*, *C. glabrata*, *C. tropicalis*, *C. krusei*, and *C. parapsilosis*) using the microdilution method (the MICs ranged from 30 to 0.4 µL/mL) [6].

### 3.2. Erigeron annuus L.

*Erigeron annuus* (L.) (commonly known as the annual fleabane or daisy fleabane) is usually an annual herb, but sometimes grows as a biennial, blooming with white petals and yellow middles in the flowers. Previously, this species was named most frequently as *Aster annuus.*

*E. annuus* has many synonyms [1]. It is an indigenous weed from eastern North America. The native distribution of *E. annuus* extends from eastern Canada to central USA and the eastern United States, including Florida, Louisiana, Mississippi, and other states [2]. The species was introduced in many European countries (including the Baltic states), some states of USA (California, Oregon, and Washington), Asia (India, Japan, Kazakhstan, Kirgizstan, Korea, Nepal, Vietnam, etc.), and some islands such as Kuril, Newfoundland, Corse, Réunion, Sicilia, Ireland, etc. [2]. *E. annuus* possesses a number of biological properties that facilitate its ability to invade and adapt to a wide range of environmental conditions. Annual fleabane is a harmful invasive weed to the natural flora of numerous countries and represents a considerable risk to agriculture.

#### 3.2.1. Compositional data of EOs of *Erigeron annuus* L.

Data of *Erigeron annuus* L. EOs (published during the period 2000–2025) is presented in Table 2 [29,32,33,34,35]. The plant material for the studies was collected in Poland, India, and Korea. *E. annuus* is an invasive species in Poland [6], cultivated or naturalized in India [36], and widely naturalized throughout Korea [37]. In Poland, *A. annuus* often grows at the same places as *A. acris* [29].

**Figure 2:**
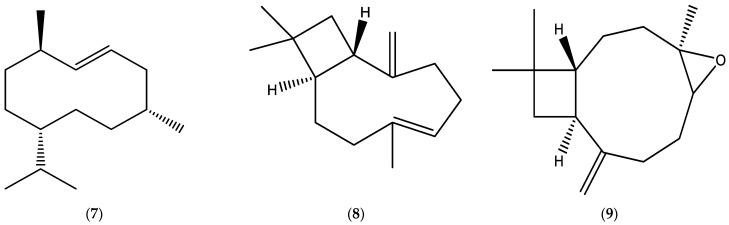
Chemical structures of germacrene D (**7**), *β*-caryophyllene (**8**), and caryophyllene oxide (**9**).

The annual fleabane EOs derived from the aerial parts have been shown to contain significant quantities of lachnophyllum ester (**6**) (Figure 1) and some sesquiterpenes, such as germacrene D (**7**) and *β*-caryophyllene (**8**), and its oxide (**9**) (Figure 2).

It should be mentioned that four new sesquiterpenoids, (7R*) opposit-4(15)-ene-1*β*,7-diol, 11-methoxyopposit-4(15)-en-1*β*-ol, 15-methoxyisodauc-3-ene-1*β*,5*α*-diol, and 10*α*-hydroxycadin-4-en-15-al, have been isolated from the areal parts of *E. annuus* [38].

#### 3.2.2. Bioactivity (Skin Regeneration and Antifungal Properties) and Toxicity (Allelopathic Effects) of EOs of *E. annuus* (L.)

The impact of the annual fleabane EO on skin-regeneration-associated responses, especially proliferation and migration, using human epidermal-keratinocytes (HaCats) was investigated by Kim et al. [35]. Results of the above research indicate that the oils stimulated proliferation and migration in HaCats and increased the phosphorylation of serine/threonine-specific protein kinase (AKT) and extracellular signal-regulated kinase (ERK) 1/2; and furthermore, the EO promoted sprout outgrowth in HaCats [35]. Additionally, the EOs from the *E. annuus* herb demonstrated a moderate antifungal activity against various strains of five *Candida* fungi (MIC values varied from 30 to 1.8 μL/mL) [6]. However, significant antifungal effects of annual fleabane EOs were documented by Kumar et al. against *Fusarium oxysporum*, *Helminthosporium maydis*, *Rhizoctonia solani*, *Alternaria solani*, and *Sclerotinia sclerotiorum* (inhibition varied from 37.6 ± 1.8 to 85.5 ± 1.4%, and IC_50_ values ranged from 660.0 to 153.2 to μg/mL). Moreover, remarkable effects of the spore germination were observed for *F. oxysporum*, *Curvularia lunata*, and *Albugo candida* (the strongest activity was assessed against *F. oxysporum* for, IC_50_ = 120.7) in the above study [34].

A recent article written by Rana et al. (published in 2023) provides a comprehensive review of the phytochemistry and biological activity of various extracts (and individual constituents) from annual fleabane [9]. It was reported herein that ten particular sesquiterpenoids of eudesmane, oppositene, cadinene, and isodaucene types of skeletons were identified in this plant. Moreover, the review [9] presents the chemical structures of twenty monoterpenoids, fifty-nine sesquiterpenoids, and eleven various polyacetylenic compounds identified in different parts of *E. annuus.*

### 3.3. Erigeron bonariensis (L.)

*Erigeron bonariensis* (L.) (commonly called as the flax-leaf fleabane or hairy fleabane) is also referred to by many alternative names, including *Conyza bonariensis* (L.) Cronquist, which is the most widely recognized [1]. It is an annual or perennial herbaceous plant that can reach a height of 1–2 m; the leaves are covered with hairs, including long hairs near the apex of the bracts. Its flowers have white ray florets and yellow-center floret disk. The native geographical distribution of the species extends from Mexico to the tropical regions of South America [2]. The species has been first described in Argentina, but it is now widely distributed across the warmer regions of Europe (predominantly in the Mediterranean basin), Africa, the Caribbean and Central America, Asia, New Zealand, and all states of Australia [39,40]. Confusion between the taxonomic classifications of *C. bonariensis*, *C. canadensis*, *C. sumatrensis*, and other *Conyza* species has been identified, especially during the initial phases of seedling development [40,41,42]. Due to the distinctive reproductive system and the high efficiency of the seed dispersal mechanism, the flax-leaf fleabane spreads rapidly. The detrimental allelopathic impact of this invasive species on plant communities and soil parameters is related to the phytotoxicity of flax-leaf fleabane [43]. It is estimated that this harmful weed has the potential to cause 28–68% yield loss in key field crops, including soybean and cotton, on an annual basis [44]. One of the effective and environmentally sustainable methods of controlling the noxious weed may be the use of other EOs [45].

#### 3.3.1. Compositional Data of EOs of *Erigeron bonariensis* L.

Data of the *Erigeron bonariensis* L. EOs (published during the period 2000–2025) are presented in Table 3 [13,39,46,47,48,49,50,51,52,53,54,55,56,57,58,59,60,61,62]. The plant material for these studies was collected from the Amazon regions in Brazil, and Argentina, Venezuela, Greece, Sardinia (Italy), Tunisia, Egypt, Kenya, Pakistan, India (Uttar Pradesh and Udham Singh Nagar regions), and Vietnam.

*E. bonariensis* species is native to S. America continent, while all taxa of *Erigeron* sect. Conyza (including *E. bonariensis*) are invasive to the Mediterranean basin. Moreover, the species was introduced in African and Asian countries [2].

**Figure 3:**
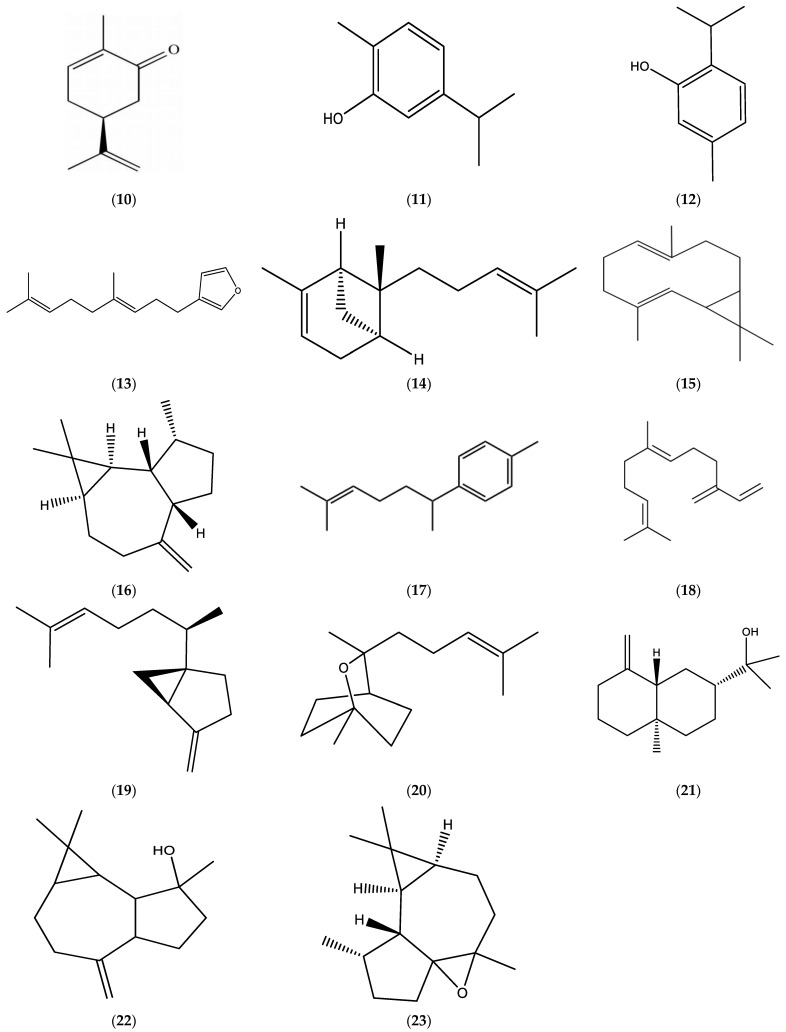
Chemical structures of carvone (**10**), carvacrol (**11**), thymol (**12**), dendrolasin (**13**), *α*-bergamotene (**14**), bicyclogermacrene (**15**), *allo*-aromadendrene (**16**), *α*-curcumene (**17**), *β*-farnesene (**18**), sesquisabinene (**19**), sesquicineole (**20**), *β*-eudesmol (**21**), spathulenol (**22**), and ledene oxide (**23**).

The EOs obtained from *E. bonariensis* (L.) were comprised of significant amounts of monoterpenes, limonene (**1**), *β*-pinene (**2**), and *β*-ocimene (**3**) (Figure 1)); oxygenated monoterpenes, matricaria (**5**) and lachnophyllum (**6**) esters (Figure 1), carvone (**10**), carvacrol (**11**), thymol (**12**), and dendrolasin (**13**) (Figure 3); sesquiterpenes, germacrene D (**7**), *β*-caryophyllene (**8**) (Figure 2), *α*-bergamotene (**14**), bicyclogermacrene (**15**), *allo*-aromadendrene (**16**), *α*-curcumene (**17**), *β*-farnesene (**18**), and sesquisabinene (**19**) (Figure 3); and oxygenated sesquiterpenes, caryophyllene oxide (**9**) (Figure 2), sesquicineole (**20**), *β*-eudesmol (**21**), spathulenol (**22**), and ledene oxide (**23**) (Figure 3).

#### 3.3.2. Bioactivity and Toxicity of *Erigeron bonariensis* EOs

A number of the oils derived from *E. bonariensis* were examined for their biological activities, including anti-inflammatory, anticancer, anti-aging, and antimicrobial activities, and toxically (insecticidal, larvicidal, nematicidal, repellent, etc.) effects [13,39,52,53,54,55,56,58,59,60,61,62,63,64,65].

An investigation was conducted by Souza et al. in which the EOs obtained from Brazilian *C. bonariensis* (L.) Cronq. were screened for anti-inflammatory activity in a mouse model of pleurisy induced by zymosan and lipopolysaccharide (LPS). The results of the study demonstrate that when the oils were administered orally, they were able to inhibit the LPS-induced inflammation, including cell migration [13]. Moreover, the EOs of the *E. bonariensis* from Egypt [59] showed a strong cytotoxicity against HepG2 (IC_50_ = 25.6 μM), and remarkable inhibitory effects of the collagenase, elastase, hyaluronidase, and tyrosinase, compared to epigallocatechin gallate (as a reference). Ferreira et al. investigated anticancer activity against human tumor cell lines (melanoma, cervical, colorectal, and leukemias) and non-tumor keratinocyte lines using an MTT assay, toxic effects using the zebrafish model, and anti-aging properties (antioxidant potential using a DCFH-DA assay, and a protection assay using the antioxidant N-acetyl-L-cysteine) [61]. In addition, cytotoxicity activity tests were performed by Araujo et al. against HeLa (cervix carcinoma), A-459 (lung carcinoma), and MCF-7 (breast adenocarcinoma) human cell lines and against normal Vero cells (African green monkey kidney); obtained IC_50_ ranged from 1.4 to 45.8 µg/mL [52]. Furthermore, the EOs demonstrated significant antimicrobial effects against *Bacillus cereus* (MIC 25–50 µg/mL) and a moderate activity against *Staphylococcus epidermidis* and *Candida albicans* (MIC 100–200 µg/mL) in the above study [52]. Antibacterial effects of the EOs from *C. bonariensis* of Indian origin were evaluated against Gram-negative bacteria *Erwinia herbicola* (Lohnis) (syn. *Pantoea agglomerans*) and *Pseudomonas putida* (Kris Hamilton) (1.42 ± 0.23 and 2.47 ± 0.9 mm of inhibition zones of these two phytopathogenic bacteria, respectively) [53]. Additionally, another study related to the antibacterial activity of the oils of Indian *E. bonariensis* was conducted by Kushwaha et al. [55]. The oils demonstrated a significant degree of activity against *Salmonella enterica*, *Pseudomonas aeruginosa*, and *Escherichia coli*, but comparatively less activity against *Staphylococcus aureus* and *Klebsiella pneumoniae* [55]. The antibacterial activity of EOs obtained from Kenyan *C. bonariensis* (L.) Cronq. was revealed using the disk diffusion method against the pathogenic *Escherichia coli* and *Salmonella typhi* strains; the MICs were determined to be 12.5% and 6.25%, respectively [56]. Antifungal effects of *C. bonariensis* EO against pathogenic *Colletotrichum musae* were investigated, and its potential application was suggested for reducing the anthracnose development in banana fruits during storage [60]. However, antimicrobial activity of the EOs of Colombian *C. bonariensis* showed activity against yeasts (*Candida parapsilosis* and *C. krusei*) and fungi (*Aspergillus flavus* and *A. fumigatus*), with MIC values of ˃ 500 µg/mL, and cytotoxic effects on the Vero cell line using an MTT assay (IC_50_ = 70 ± 16.1 μg/mL) [63]. In another study [54], antimicrobial and insecticidal properties were assessed; it was determined that the EOs exhibited moderate effects against *B. subtilis* (MIC = 125 μg/mL) and a strong larvicidal activity against the adults of *C. pipiens* mosquitoes (EC_50_ = 2.455 ppm) and against the same larvae (EC_50_ = 9.307 ppm) [54]. Furthermore, mosquito larvicidal activity was revealed against *Aedes aegypti*, *Ae. albopictus*, and *Culex quinquefasciatus* in the study by Hoi et al. [58]. However, insecticidal and nematicidal activities of the EOs from *C. bonariensis* plants collected in Togo were tested on cowpea weevil *Callosobruchus maculatus* adults (LC_50/24h_ = 1.75 μL oil/L air and 100% mortality at 3.91 μL oil/L air) and on freshly hatched second-stage juveniles of root-knot nematode *Meloidogyne incognita* (EO from the whole plant was the most effective, EC_50/72h_ = 1817 mg/L) [39]. Results of larvicidal and repellent activity tests against adult female mosquitos and the larvae of yellow fever mosquitos, *Aedes aegypti*, show that the flax-leaf fleabane EOs exhibited 40.2% repellency against mosquitoes at a tested dose of 33.3 μg/cm^2^, more than 40% repellency for 60 min at a tested dose of 330 μg/cm^2^ in time-span bioassays, and that the larvae were very susceptible to the EOs upon 48 h. exposure (LC_50_ = 26.0 mg/L) [62].

Recently, two review articles were published by Opiyo et al. and Mahanur et al., which examined the chemical composition and biological activity of the EOs of *C. bonariensis* [64] and the red listed South African plant *Erigeron bonariensis*: var. *microcephala* Cabrera (EB), respectively [65].

Given the sufficient quantity of published data, a statistical analysis was conducted to examine the relationships between the compositional variability and bioactivity of *C. bonariensis* EOs. Refer to the illustration presented in the Supplementary Material (Figure S1).

### 3.4. Erigeron breviscapus (Vaniot) Hand.-Mazz.

*Erigeron breviscapus* (also known as *Herba Erigerontis* or *Lamp Chrysanthemum)* has several synonyms, including *Erigeron dielsii* and *Erigeron praecox.* It is a perennial herb growing primarily in the temperate regions in Asian, and is a native species in Tibet and South-Central and South-East parts of China (Yunnan, Hunan, Guangdong, Guangxi, Guizhou, and Sichuan provinces) [2]. *E. breviscapus* is 5–50 cm in height, has woody and thick rhizomes, the stem is upright with a few branches in the middle, and the whole plant is covered with bristles or mixed with glandular hairs; the leaves are mainly concentrated at the base and are in the shape of a rosette; the flower head is solitary [66]. Usually, this species grows at an altitude of between 1,700 and 3,000 m in open hillside grassland and at the edges of forests.

#### Compositional Data of EOs of *Erigeron breviscapus* and Their Bioactivity

To date, there is a paucity of literature concerning EOs of *Erigeron breviscapus*. A detailed review of its phytochemistry, pharmacology, clinical applications, and toxicology was published by Wu et al. [66]. The whole plant has been used in Chinese medicine for more than 600 years due to its broad range of pharmacological effects, including cerebrovascular, cardiovascular, anti-diabetic, anti-inflammatory, antioxidant, neuroprotective, etc., activities [14,15,66]. *β*-Pinene, 1,8-cineole, thymol, *α*-terpineol, borneol, eugenol, and nerolidol were found to be the main characteristic terpenoids, and the chemical structures of ten volatiles are presented herein [66].

### 3.5. Erigeron canadensis L.

*Erigeron canadensis* has numerous synonyms (at least 35, with *Conyza canadensis* (L.) being the most common), and is commonly called as a horseweed or Canadian fleabane. The species is considered to be an annual or perennial weed, indigenous to both American continents and widespread around the world [1,2]. It can grow to be 1.5 m tall, has hairy stems, the leaves are long (2–10 cm) with toothed margins and dense inflorescences, and each individual flower has white or light purple florets and yellow-center disks. *E. canadensis* is an invasive weed in many countries and exhibits resistance to multiple herbicides, thereby causing considerable damage to agricultural land [45].

#### 3.5.1. Compositional Data of EOs of *Erigeron canadensis* L.

The main data of *E. canadensis* EOs (published during the period 2000–2025) are presented in Table 4 [48,53,58,62,67,68,69,70,71,72,73,74,75,76,77,78,79,80,81,82,83]. The plant material for these studies was collected in Brazil (where *E. canadensis* is a native species), in many European countries (Poland, France, Italy, Spain, Greece, Hungary, Belgium, Bulgaria, and Lithuania), and in Ethiopia, Turkey, Iran, Jordan, Pakistan, India, China, Korea, and Vietnam, where the species is invasive [2].

**Figure 4:**
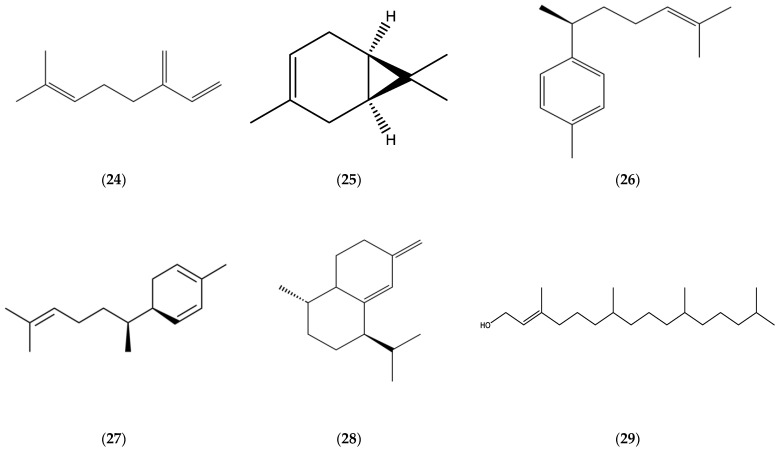
Chemical structures of myrcene (**24**), *δ*-3-carene (**25**), *ar*-curcumene (**26**), zingiberene (**27**), *epi*-bicyclosesquiphellandrene (**28**), and phytol (**29**).

Monoterpenes, limonene (**1**), *β*-pinene (**2**)**,** *β*-ocimene (**3**) (Figure 1), myrcene (**24**), and *δ*-3-carene (**25**) (Figure 4); oxygenated monoterpenes, matricaria (**5**) and lachnophyllum (**6**) esters (Figure 1); sesquiterpene hydrocarbons, germacrene D (**7**), *β*-caryophyllene (**8**) (Figure 2), *α*-bergamotene (**14**), *β*-farnesene (**18**) (Figure 3), *ar*-curcumene (**26**), zingiberene (**27**), and *epi*-bicyclosesquiphellandrene (**28**) (Figure 4); oxygenated sesquiterpenes, caryophyllene oxide (**9**) (Figure 2) and spathulenol (**22**) (Figure 3); and a diterpenoid, phytol (**29**) (Figure 4) were determined among the major constituents present in the EOs of *Erigeron canadensis* L. (*Conyza canadensis* (L.) Cronq.).

#### 3.5.2. Bioactivity and Toxicity of *Erigeron canadensis* (L.) EOs

Strong cytotoxic activity was revealed for the EOs obtained from the aerial part of Korean *E. canadensis* L. in an MTT assay using HaCaT keratinocyte cells (IC_50_ = 0.027 µg/mg) [72]. Furthermore, its substantial antitumoral potential was noticed for horseweed leaf EOs containing high amounts of limonene against neoplastic cell lines K562 (leukemia) and NCI-ADR/RES (ovary with multidrug resistance phenotype), with TGI values of 16.8 and 19.0 mg/mL, respectively [81]. Moreover, under the impact of the oils (rich in limonene, up 65.7%) of Chinese origin, the cell viability of the normal liver cell lines L02 and the human cervical cancer cell lines HeLa dramatically declined [84].

The horseweed EOs were demonstrated to possess a weak capacity to inhibit the growth of some phytopathogenic fungi, such as *Rhizoctonia solani* Kuhn, *Fusarium solani* (Mart.) Sacc., and *Colletotrichum lindemuthianum* (Sacc. & Magn.) Briosi & Cav. [44]. On the other hand, the Canadian fleabane EOs, comprised of more than 50% of limonene, exhibited a significant broad spectrum of antimicrobial effects against some food-borne pathogens and fungi, especially on *Alternaria panax* Whetz, *Rhizoctonia solani*, and *Shigella dysenteriae* [85]. Furthermore, the above-mentioned oils possessed inhibition properties on germination and the seedling growth of plants, and can enhance the length of the *Daucus carota* var. *sativa* L. roots. Moderate to strong fungicidal effects against the reference fungal strains, and fungal strains isolated from patients (*Aspergillus*, *Candida*, *Cryptococcus*, *Rhodotorula*, and *Trichophyton*), except against *A. fumigatus*, were revealed by Veres et al. [75]. The highest zones of inhibition were observed on *Cryptococcus neoformans* and *Trichophyton interdigitalis* in the above study. However, the antimicrobial activity of horseweed EOs was assayed against three bacteria (*Salmonella enteritidis*, *Staphylococcus aureus*, and *Pseudomonas aeruginosa*) and three fungi (*Alternaria alternata*, *Aspergillus niger*, and *Penicillium digitatum*) applying the modified disk volatilization method [76]. Moreover, the antibacterial tests of Indian horseweed EOs showed 1.50 ± 0.70 and 8.17 ± 0.88 mm of inhibition zones of two Gram-negative bacteria *Erwinia herbicola* (Lohnis) and *Pseudomonas putida* (Kris Hamilton), respectively [53]. Screening of the EOs against both Gram-positive and Gram-negative bacteria and one strain of fungus using the broth microdilution method showed the highest antibacterial activity of the oils from aerial parts against *Escherichia coli* RSKK 234 (MIC = 0.039 μg/mL) and *Candida albicans* ATCC 10231 (MIC = 0.078 μg/mL) [79]. The EOs from *C. canadensis* growing wild in the Kashmir valley (India) exhibited significant antibacterial activity against the tested Gram-positive and Gram-negative food-borne bacteria (MIC values ranged from 12.00 to 40.00 μg/mL), and the strongest inhibitory effects were determined against *Candida albicans* and *Candida parapsilosis*, with MICs of 2.50 and 1.80 μg/mL, respectively [80]. Also, the EO investigated above showed an effective antioxidant potential using DPPH and hydroxyl radical scavenging assays [80].

Furthermore, the allelopathic properties of horseweed EOs on seed germination of the receptor plants (*Brassica chinensis* Linn., *B. pekinensis* Rupr., *Triticum sestivum* Linn., and *Sorghum bicolor* (Linn.) Moench) were assessed in the studies by Liu et al. [73,74]. The significant insecticidal potential of the fleabane EOs against the first–fourth instar larvae and pupae of *Aedes albopictus* and *Culex quinquefasciatus* was revealed using the immersion and fumigation methods [78]. Horseweed EOs demonstrated repellent and larvicidal activity against *Aedes aegypti*, *Ae. albopictus*, and *Culex quinque* [58,82]. The EOs of *E. canadensis* from Pakistan exhibited 41.7% repellence against mosquitoes (at a tested dose of 33.3 μg/cm^2^), and the larvae of *Ae. aegypti* were susceptible to the tested oils (LC_50_ = 35.7 mg/L, upon 48 h exposure) in time-span bioassays [62].

A statistical analysis was conducted on the available published data in order to examine the compositional variability and bioactivity of *E. canadensis* EOs. Refer to the visual depiction of a biplot provided in the Supplementary Material (Figure S2).

### 3.6. Erigeron floribundus (Kunth) Sch.Bip. (Erigeron sumatrensis)

*Erigeron floribundus* (commonly known as Bilbao fleabane, Asthma weed, tall fleabane, many-flowered fleabane, and Bilbao’s Erigeron) has many synonyms, and the most frequent is *Conyza floribunda* Kunth, *Conyza sumatrensis* var. *floribunda* (Kunth), and *Conyza albida* Willd. ex Spreng. [2]. *C. albida* and *C. floribunda* are treated as synonyms of *C. sumatrensis.* The species of *Conyza* showed differentiation in the inflorescence topology and the structural features of the capitulum. The stem and leaf cover’s trichomes are heterogeneous in *C. sumatrensis* var. *sumatrensis*, and it has one set of uniform abundant short hair on both faces of the leaves. *C. sumatrensis* var. *floribunda* possesses a hirsute pubescence with few trichomes above the rib of the stem and margin of the leaf, few trichomes in the central nervation of the phyllary, and biseriate glandular hair in the corolla apical region of the flowers. *C. sumatrensis* var. *floribunda* presents short hair on both faces of the leaf [49].

*E. floribundus* is an herbaceous plant, up to 1.5 m in height, with pubescent, lanceolate leaves and flowers in yellowish panicles. It is an annual or biennial herb of American origin, a persistent invader spreading throughout the world, and a difficult weed to control.

#### 3.6.1. Compositional Data of EOs of *Erigeron floribundus*

**Figure 5:**
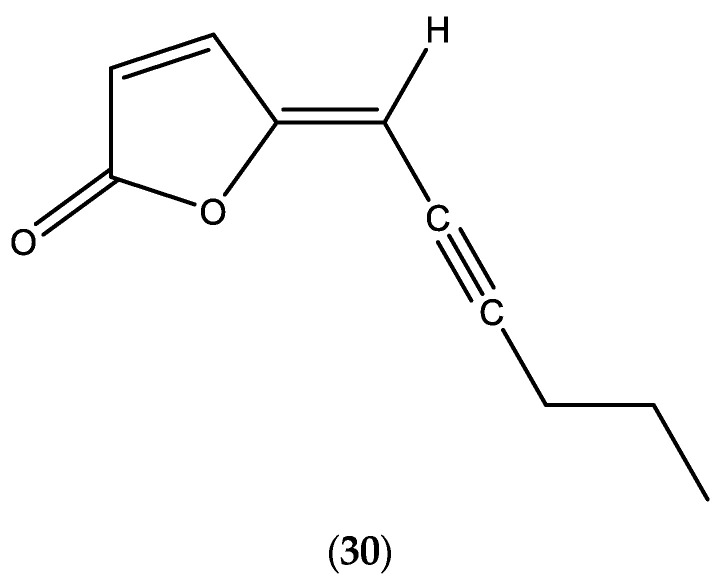
Chemical structure of lachnophyllumlactone (**30**).

The main data of *Erigeron floribundus* (*E*. *sumatrensis*) EOs, published during the period 2000–2025, are presented in Table 5 [48,49,86,87,88,89,90,91,92]. Specific constituent, lachnophyllumlactone (**30**) was identified the EOs (Figure 5). In order to avoid any confusion due to the taxonomic attribution of the *Erigeron* species, identification of the species’ names performed by the authors is included in Table 5. The plant material utilized in these studies was collected in Argentina (where it is a native species), Greece, Tunisia, Cameroon, Côte d’Ivoire, India, and Pakistan, where the species was introduced [2].

Two new sesquiterpenoids, (7R*) opposit-4(15)-ene-1*β*,7-diol and 15-methoxyisodauc-3-ene-1*β*,5*α*-diol, have been isolated from *E. sumatrensis* plants for the first time [12].

#### 3.6.2. Bioactivity and Toxicity of *Erigeron floribundus* (L.) EOs

The EOs obtained from *E. floribundus* growing in Cameroon exhibited a broad spectrum of fungicidal effects against *Trichophyton rubrum*, *Trichophyton mentagrophytes*, *Candida albicans*, and *Cryptococcus neoformans* [87]. The flower’s EO was found to be more active than the leaf’s oil. It was revealed that *C. albicans* was the most sensitive fungus (MIC = 2.25 µL/mL for both oils), and the MIC values for other fungi ranged from 12.5 to 8.5 µL/mL [87]. In addition, the EOs of aerial parts of Bilbao fleabane from Cameroon showed a good inhibition activity against *Staphylococcus aureus* (MIC = 512 µg/mL), against the NadD enzyme from *S. aureus* (IC_50_ = 98 µg/mL, and with no effects on mammalian orthologue enzymes), and on *T. brucei* proliferation (IC_50_ = 33.5 µg/mL) in another study [91]. However, the above-mentioned oils demonstrated a strong cytotoxicity on HCT 116 colon carcinoma cells (IC_50_ = 14.89 µg/mL) and remarkable antioxidant properties (tocopherol-equivalent antioxidant capacity, TEAC = 411.9 μmol·TE/g) [91].

The *E. floribundus* EO was screened against ten human pathogenic bacteria and fungi [89]. The oil was found more active against the tested fungal strains, with MIC values of 0.41 ± 0.18, 0.7 2± 0.47, 0.36 ± 0.23, 0.45 ± 0.28, 0.57 ± 0.59, and 0.88 ± 0.63 mg/mL against *Staphylococcus aureus*, *Escherichia coli*, *Candida albicans*, *Aspergillus niger*, *Saccharomyces cerevisiae*, and *Penicillium chrysogenum*, respectively [89]. The oils were evaluated for antibacterial, antifungal, and allelopathic activities [90]. The results indicate that the leaf’s oil exhibited significant antibacterial activity against *Enterococcus faecalis*, *Staphylococcus aureus*, and *Proteus mirabilis;* and that the *C. sumatrensis* oils isolated from the aerial parts presented high mycelia-growth inhibition of *Candida albicans* and the filamentous fungi tested. Moreover, the EOs of the different plant parts inhibited the shoot and root growth of *Raphanus sativus* (radish) seedlings. Indeed, the inhibition of the hypocotyl growth varied from 28.6 to 90.1% and that of the radicle from 42.3 to 96.2%. Furthermore, the allelopathic potential of the oil and its fractions was evaluated using two sensitive indicators, *Avena sativa* and *Spirodella polyrhiza* [86]. Furthermore, Azeem et al. assessed a pesticidal of potential of *C. sumatrensis* EOs against grain pests, the red flour beetle *Tribolium castaneum* (toxicity on adult population: LD_50_ = 33.91 mg/10 g rice and LD_90_ *=* 126.9 mg/10 g rice; and fumigation toxicity at 72 h. exposure: LD_50_ = 6.620 mg/10 g rice and LD_90_ *=* 15.83 mg/10 g rice;) and the mold *Aspergillus flavus* (16.5 mm zones of inhibition) [92].

Guetchueng et al. conducted in 2023 an extensive review of the phytochemistry, pharmacology, toxicology and traditional uses of *E. floribundus* [93].

### 3.7. Erigeron graveolens L.

*Erigeron graveolens* is a basionym for a number of other names, the most common of which are listed below. These include *Dittrichia graveolens* (L.) *Greuter, Inula graveolens* (L.) Desf., *Solidago graveolens* (L.) Lam., *Paniopsis graveolens* Raf., *Cupularia graveolens* (L.), and *Pulicaria graveolens* (L.) [2,94]. It is an annual plant, which is commonly known as stinkwort or stinking fleabane, a branched subshrub that grows up to 1.30 m tall and possesses an aromatic camphor-like odor. The native geographical distribution of this species extends from Southern Europe (the Mediterranean region) through to North Africa and as far as Western Asia and the Western Himalayas in Pakistan.

#### 3.7.1. Compositional Data of *Erigeron graveolens* (L.) EOs

A detailed and very informative review concerning the chemistry and bioactivity of *Dittrichia graveolens* (L.) Greuter was published in 2022 [23]. Consequently, the subsequent data of EOs are presented in Table 6 [95]. *E. graveolens* is an indigenous species in Tunisia [2].

**Figure 6:**
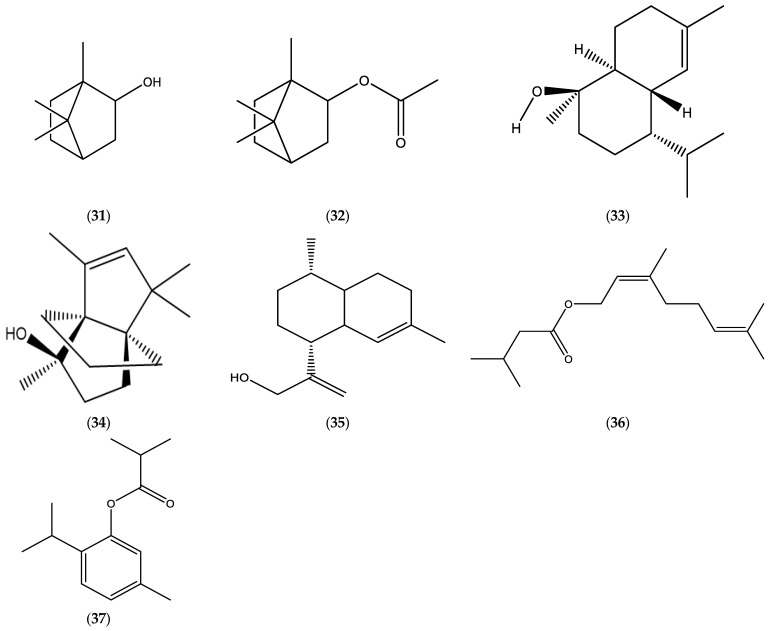
Chemical structures of borneol (**31**), bornyl acetate (**32**), *τ*-cadinol (**33**), modhephen-8-*β*-ol (**34**), *cis*-arteannuic alcohol (**35**), neryl isovalerate (**36**), and thymol isobutyrate (**37**).

The main constituents of the EOs from aerial parts of *E. graveolens* (identified as *Inula graveolens* (L.) or *Dittrichia graveolens* (L.) Greuter)) were determined to be borneol (**31**), bornyl acetate (**32**), β-caryophyllene (**8**), caryophyllene oxide (**9**), and *τ*-cadinol (**33**) (Table 2, Figure 6) [23,95,96]. The root oils under consideration were characterized by the following components: sesquiterpenoids, modhephen-8-*β*-ol (**34**) and *cis*-arteannuic alcohol (**35**); and monoterpene esters, neryl isovalerate (**36**) and thymol isobutyrate (**37**) (Table 6, Figure 6).

#### 3.7.2. Bioactivity and toxicity of *E. graveolens* (*Inula graveolens* (L.)) EOs

To the best our knowledge, there is an absence of available data concerning the biological properties of *E. graveolens* EOs. For the first time, in 2023, Mustapha et al. evaluated the repellence and contact toxicity properties of EOs from aerial parts and roots of *Inula graveolens* against adults of stored-product beetle *Tribolium castaneum*. The oils displayed strong repellence effects after 2 h of exposure (up to 90.0 % at 0.12 μL/cm^2^); and the LD_50_ values were found to be 7.44% and 4.88%, respectively, for the root and aerial parts EOs [95].

### 3.8. Erigeron incanus Vahl (Erigeron leucophyllus (Sch. Bip. ex A. Rich.) Schweinf.)

*Erigeron incanus* Vahl (called by common names such as wooly daisy, wooly fleabane or, wooly erigeron) is known by several synonyms (the most common being *Erigeron leucophyllus* and *Conyza incana* Willd.) [1]. *E. incanus* is a native species to Eritrea, Ethiopia, Somalia, Yemen, and Saudi Arabia, where it is found in deserts and dry shrublands [2]. It is a distinctive, branched, dense, gray-tomentose undershrub up to 50 cm tall with very leafy stems. It is widespread and frequent on steep, bare rocky hillsides from 1600 to 3100 m on the escarpment and high plateau in Yemen [24].

#### 3.8.1. Compositional Data of EOs of *Erigeron incanus* Vahl

**Figure 7:**
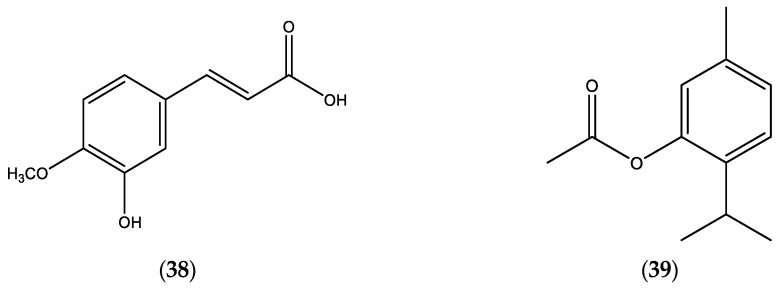
Chemical structures of 3-hydroxy-4-methoxy cinammic acid (**38**) and thymol acetate (**39**).

Limited data of *E. incanus* EOs (published during the period 2000–2025) are documented in Table 7 [24]; and a chemical structure of the main constituents, 3-hydroxy-4-methoxy cinammic acid (**38**) and thymol acetate (**39**), in the EOs of *E. incanus* are presented in Figure 7.

#### 3.8.2. Bioactivity of EOs of *Erigeron incanus*

Antimicrobial activity of the EO of *E. incanus* from Yemen was evaluated against two Gram-positive (*Staphylococcus aureus* and *Bacillus subtilis*) and two Gram-negative (*Escherichia coli*, *Pseudomonas aeruginosa*) bacteria and on *Candida albicans* using the disk diffusion method [24]. The oil demonstrated strong fungicidal effects (MIC = 40 μg/mL against *C. albicans*) and bactericidal activity against *B. subtilus* and *S. aureus*, with MIC(s) values of 150 μg/mL and 234 μg/mL, respectively. Moreover, the oil showed a strong antioxidant activity in a DPPH radicals scavenging assay (IC_50_ = 5.2 μg/mL) [24]. EOs from the seeds of wild *E. incanus*, collected in Yemen (Taiz-Hojariah region), exhibited antimicrobial (moderate effects against *Proteus vulgaris* bacterial and *Fusariumoxy sporium* fungal strains) and antioxidant properties [97,98].

### 3.9. Erigeron mucronatus DC

*E. mucronatus* (common name Mexican fleabane) is a synonym of *Erigeron karvinskianus.* However, the perennial species is known by other synonyms, the most frequent being *Erigeron karvinskianus* var. *mucronatus* (DC.) Asch. or *Erigeron trilobus* Sond. [2]. The native range of this species is the subtropical regions in Central America, Colombia, Mexico, and Venezuela. It is naturalized in many other world parts of Africa, Europe, Australia, Chile, and the west coast of the USA. Moreover, it is also cultivated for the purpose of its distinctive daisy-like blooms. It is an annual herb reaching about 3 m in height with either white- or pink-colored flowers [25].

#### 3.9.1. Compositional Data of Erigeron mucronatus (Erigeron karvinskianus) EOs

The main chemical composition of the EOs of *Erigeron mucronatus* are presented in Table 8 [25,34]. The plant material utilized in these investigations was collected in India, where the species is not indigenous [2].

#### 3.9.2. Bioactivity of Erigeron mucronatus (Erigeron karvinskianus) EOs

Awen et al. reported that *E. mucronatus* EOs exhibited a strong antibacterial activity against *Staphylococcus aureus* and *Escherichia coli* strains and a moderate activity against *Pseudomonas aeruginosa*. Moreover, the genotoxicity of the oil was evaluated in AB blood serum in the above study [25]. Antifungal effects of the EOs from the Himalayan *Erigeron* species (*E. mucronatus* and *E. karwinskianus)* against the tested fungi in the growth inhibition range of 40.4–83.9% (with IC_50_ values ranging from 88.8 to 602.7 μg/mL) were revealed, and the most significant inhibition of spore germination was determined for *Fusarium oxysporum*, *Curvularia lunata,* and *Albugo candida* [34].

### 3.10. Erigeron multiradiatus (Lindl. ex DC.) Benth

*Erigeron multiradiatus* (commonly known as Himalayan fleabane) is a perennial or annual Asian species with violet or dark-purple flowers native to the eastern and western parts of the Himalayas (Pakistan, Afghanistan, Nepal, Tibet, Bhutan, etc.). The species has several synonyms. It grows primarily in the subalpine or subarctic regions of South–Central Asia (Iran), Afghanistan, and South–Central China [2]. It is a beautiful plant with an erect hairy stem up to 40 cm; the leaves are ovate or lance-like, pointed or blunt, and dented at the tip. Flower-heads vary in size 1.5–5 cm across; the central disk is yellow. Distribution of this species is from 2600 to 4400 m in the Himalayas [36].

#### Compositional Data of *Erigeron multiradiatus* EOs and Their Activity

It was reported that the EO obtained from the aerial parts of *Erigeron multiradiatus* (Lindl. ex DC.) Benth growing wild in the central Himalayan region (Uttarakhand, India, where the species is native) and comprised by the predominant constituents, *trans*-2-*cis*-8-matricaria-ester (77.8%) and *cis*-lachnophyllum ester (11.0%), exhibited remarkable leishmanicidal effects against *Leishmania donovani* promastigotes and intracellular amastigotes [99].

### 3.11. Erigeron philadelphicus L.

*Erigeron philadelphicus* (commonly known as the Philadelphia fleabane or fleabane daisy) is a perennial herb that is native to the subarctic regions of North America and the USA [2]. However, this species was introduced in Europe (France, Germany, Great Britain, Italy, etc.), Asia (China, Japan, Korea, etc.), and some islands (Corse, Newfoundland, and Mauritius). The optimal climatic conditions of the plants are temperate regions. *E. philadelphicus* has been identified as an ecologically destructive invasive species in numerous countries. The fleabane daisy grows along roadsides and in fields and woodlands. Its yellow center with a large number of very fine white ray flowers is its best identifier [100].

#### Compositional Data of *Erigeron philadelphicus* EOs and Their Activity

A sesquiterpenoid, 6*β*,14-epoxyeudesm-4(15)-en-1*β*-ol, and a diterpenoid, philadelphinone, were isolated for the first time from the aerial parts of *E. philadelphicus* plants [38]. Moreover, several other new terpenoids and related compounds were identified in *E. philadelphicus* by Yaoita et al. [101]. No available date concerning the biological properties of *E. philadelphicus* EOs has been reported over the past 25 years.

### 3.12. Erigeron strigosus Muhl. ex Willd (Erigeron ramosus)

The species has many synonyms (known by the common names daisy fleabane, prairie fleabane, vergerette rude, or common eastern fleabane), at least 22, including *Erigeron annuus* var. *ramosus* (Walter) Hyl. [2,4]. *E. strigosus* is a native species to Canada and the USA, growing in temperate regions. Additionally, the species has become an invasive plant in Newfoundland Island, many parts of Asia (China, Tibet, Japan, Korea, and Sakhalin), and Europe (Germany) [2]. This species is categorized as an annual, biennial, or short-lived perennial, producing a multitude of flower heads. It is 30–70 cm tall, fibrous-rooted; the stems are erect or ascending, eglandular; the leaves are basal and cauline. Ray florets are of corollas white, pinkish, or bluish [4].

Two species, namely *E. strigosus* Muhl. ex Willd and *E. annuus* (L.) Pers. were documented as invasive weeds in Lithuania (in 2004) [102].

#### 3.12.1. Compositional Data of *Erigeron strigosus* (*Erigeron ramosus*) EOs

**Figure 8:**
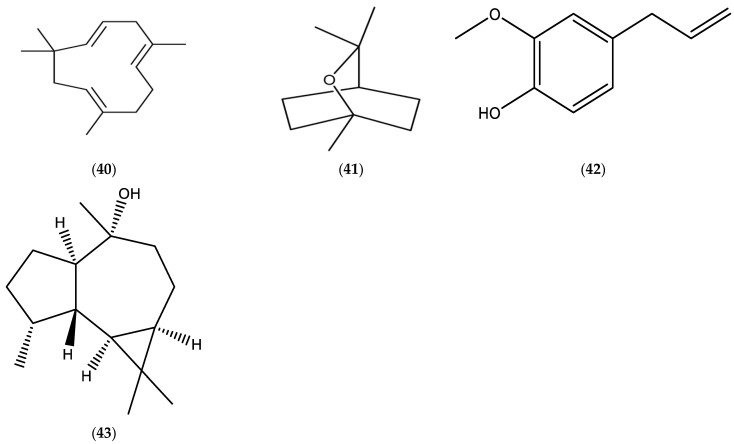
Chemical structures of *α*-humulene (**40**), 1,8-cineole (**41**), eugenol (**42**), and globulol (**43**).

There is a lack of available data related to the investigations of *Erigeron strigosus* (*Erigeron ramosus*) EOs and their properties. Limited data of *E. ramosus* EOs (published during the period 2000–2025) are documented in Table 9 [103], and a chemical structure of the main constituents, *α*-humulene (**40**), 1,8-cineole (**41**), eugenol (**42**), and globulol (**43**), in the EOs are presented in Figure 8.

#### 3.12.2. Biological Activity of *Erigeron strigosus* (*Erigeron ramosus*) EOs

The antibacterial activity of the EO from *E. ramosus* (WALT.) B.S.P. plants growing in Korea was determined against fourteen (seven g-positive and seven g-negative) foodborne bacteria, and the strongest activity was revealed against seven Gram-positive bacteria stains of *Staphylococcus aureus*, *Listeria monocytogenes*, and *Bacillus subtilis*, and four Gram-negative bacteria lines (*Pseudomonas aeruginosa*, *Enterobacter aerogenes*, and *Escherichia coli*) with MIC values ranging from 62.5 to 500 µg/mL [103].

### 3.13. Erigeron speciosus (Lindl) DC.

*Erigeron speciosus* (commonly known as aspen fleabane, garden fleabane, or Oregon fleabane) has many synonyms (at least 16). It is a perennial plant with a native population in the western parts of Canada to the northwest of Mexico. The species was introduced in some European countries (Great Britain, Sweden, Baltic States, France, Germany, Ukraine, etc.), Uzbekistan, and Indonesia (Jawa island) [2]. It is perennial, usually 30–80 cm tall, rhizomatous, fibrous-rooted, stems erect, leaves basal (usually withering by flowering), and cauline. Ray florets range from a blue to lavender color. Preferring growing locations are gravelly or loamy soil, prairies, and pine, pine–fir, spruce–fir, aspen–spruce forests and their edges [4].

#### Compositional Data of *Erigeron speciosus* EOs and Their Activity

Steam distillate from the aerial parts of *E. speciosus*, containing appreciable amounts of matricaria esters, was tested against strawberry plant pathogenic fungi *Botrytis cinerea*, *Colletotrichum acutatum*, *Colletotrichum_fragariae*, and *C. gloeosporioides.* Additionally, its molluscicidal activity was assessed against the intermediate host snail *Planobdella trivolvis* in a study [104].

### 3.14. Erigeron sublyratus Roxb. ex DC.

*Erigeron sublyratus* has several synonyms (including *Erigeron hirsutus* Wall., *Conyza graveolens* Wall. ex DC.) [2]. It is an annual herb native to the Indian Subcontinent (the Eastern Himalayan region, Nepal, India, and Sri Lanka) and to the Indo-China region (Myanmar, Thailand, and Vietnam). The plant is an erect, aromatic, branchlets hirsute, annual herb. Its leaves alternate from oblanceolate to spathulate. Ray florets are numerous, of a purplish color, and disk florets are yellowish. Preferred growing site is plains [36].

#### 3.14.1. Compositional Data of *Erigeron sublyratus* EOs

As demonstrated in Table 10, the available data concerning *E. sublyratus* EOs is extremely limited during last quarter of a century [105].

#### 3.14.2. Biological Activity of *Erigeron sublyratus* EOs

In a paper published in 2025, the composition of *E. sublyratus* EOs and their anti-inflammatory and cytotoxic properties were investigated for the first time [105]. The EOs from aerial parts inhibited nitric oxide (NO) production on LPS-induced RAW 264.7 cells (IC50 = 1.41 ± 0.10 μg/mL). In addition, both the root and aerial EOs were found to display cytotoxic activity against MCF-7, SK-LU-1, and HepG2 cell lines [105].

## 4. Discussion

A compositional revision of various EOs obtained from fourteen *Erigeron* sp. from various countries worldwide revealed significant interspecies as well as intraspecies variability. Chemical variability (both quantitative and qualitative) was determined between different plant organs and at various phenological stages of revised *Erigeron* sp. [25,28,29,33,39,50,51,68,75,79,83,95]. Furthermore, the preparation method, technique, and duration employed in the extraction process are all of paramount importance to the composition of the final product.

A sufficiently extensive list of the most prevalent characteristic compounds was established. Limonene, *δ*-3-carene, matricaria ester, lachnophyllum ester, germacrene D, *β*-caryophyllene, *β*-farnesene, *α*-bergamotene, *allo*-aromadendrene, etc., were determined in the oils from the *E. acris*, *E. annuus*, *E. bonariensis*, *E. canadensis*, *E. floribundus*, *E. mucronatus*, and *E. speciosus* plants. Specific constituents, such as monoterpenoid, dendrolasin; diterpenene, neophytadiene and manool (a labdane type diterpenoid)*;* 2,6,7,7*α*-tetrahydro-1,5-dimethyl-1H-indene-3-carboxaldehyde; and sesquiterpenoid, ledene oxide were identified in the EOs of *E. bonariensis* L [47,51,56,59]. Appreciable quantities of the rare constituents, 2,3-dimethyl-4(3H)-quinazolinone and lachnophyllumlactone, were identified in the oils of *E. canadensis* and *E. sumatrensis*, respectively [74,86]. Other predominant characteristic constituents, such as borneol, bornyl acetate, modhephen-8-*β*-ol, *cis*-arteannuic alcohol, neryl isovalerate, thymol isobutyrate, and *τ*-cadinol, were determined in the *E. graveolens (Inula graveolens*) oils [95]. A paucity of data concerning *E. incanus* EOs was revealed, and 3-hydroxy-4-methoxy cinammic acid and thymol acetate were found to be the major ones [24]. The EOs from *E. multiradiatus* and *E. sublyratus* are characterized mostly by matricaria and lachnophyllum esters [99,105]. The available data on EOs of *E. ramosus* is limited, but the main constituents are known to be *α*-humulene, 1,8-cineole, eugenol, and globulol [103]. The species *E. breviscapus* has a very long history in Chinese ethnopharmacology and is cultivated in significant amounts; therefore, a section regarding its EOs was included, despite the fact that available data regarding volatile secondary metabolites of *E. breviscapus* is very limited [14,15,66].

The different classes of compounds present in the EOs are responsible for their numerous pharmacological and toxic activities. EOs are comprised of a mixture of numerous different compounds with different mechanisms of actions that may work in synergy. It is erroneous to assert that the responsibility for the biological activity lies exclusively with the main constituents. It is evident that the impact of other compounds present in the oils in quantities that may be negligible must not be overlooked. However, in order to simplify a verification of the correlation between EO composition and activity, it is necessary to consider the factors below, and I will highlight the most promising pharmacological and toxic effects of the EOs and correlate these properties with the principal constituents only. The antimicrobial properties of the EOs of various *Erigeron* species were mostly investigated. Antifungal and antibacterial effects against various strains were exhibited by the oils of *E. acris*, *E. annuus*, *E. bonariensis*, *E. canadensis*, *E. floribundus*, *E. incanus*, *E. mucronatus*, *E. speciosus*, and *E. ramosus*. These oils were characterized by appreciable quantities of limonene, *β*-pinene, 1,8-cineole, carvacrol, thymol acetae, *β*-eudesmol, 2,6,7,7*α*-tetrahydro-1,5-dimethyl-1H-indene-3-carboxaldehyde, caryophyllene and its oxide, *allo*-aromadendrene, *α*-humulene, farnesene isomers, carvacrol, globulol, or eugenol. The EOs of *E. acris*, *E. annuus*, *E. bonariensis*, *E. canadensis*, *E. floribundus*, *E. mucronatus*, *E. multiradiatus*, *E. sublyratus*, and *E. speciosus* containing appreciable amounts of matricaria and lachnophyllum esters exhibited strong anticancer, antimicrobial, anti-inflammatory, leishmanicidal, or larvicidal and repellent activities [6,9,25,29,34,39,61,62,64,65,75,79,80,86,87,90,92,93,99,104]. Moreover, repellence and contact toxicity against adults of *T. castaneum* was related to the high content of borneol, bornyl acetate, caryophyllene derivatives, *τ*-cadinol, modhephen-8-*β*-ol, and *cis*-arteannuic alcohol in *I. graveolens* oils [95]. Cytotoxicity was determined due to the presence of limonene, *δ*-3-carene, *α*- and *β*-farnesene, (*E*)-*β*-ocimene, ledene oxide, sesquiphellandrene, and dendrolasin in the fleabanes (*E. acris*, *E. bonariensis*, *E. canadensis*, *E. floribundus*, and *E. sublyratus*) EOs [6,52,59,61,63,72,81,84,91,105]. Skin-regeneration and antifungal properties were related to germacrene D in *E. annuus* oils [35]. The anti-inflammatory effects were exhibited because of the high amounts of limonene and (*E*)-*β*-ocimene in *E. bonariensis* oils [13], and, owing to lachnophyllum ester, germacrene D and *trans-β*-ocimene in *E. sublyratus* EOs [105].

A sufficient amount of published data concerning *C. bonariensis* and *E. canadensis* EOs allowed us to apply a statistical data analysis and examine the relations between compositional variability and their activity. PCA and biplots demonstrate the correlations between major components of the EOs *E. bonariensis* and *E. canadensis* their biological activities, respectively (Supplementary Material, Figures S1 and S2). Cytotoxicity of *E. bonariensis* EOs against HepG2 and the inhibitory effects of collagenase, elastase, hyaluronidase, and tyrosinase was related to *α*-farnesene, *α*-maaliene, dendrolasin, and ledene oxide. *β*-Ocimene, sesquiphellandrene, and *β*-farnesene were responsible mostly for the cytotoxic effects of the oils against HeLa, A-459, and MCF-7 human cell lines, as well as against normal Vero cells; and antimicrobial effects against *B. cereus*, *S. epidermidis*, and *C. albicans.* Matricaria and lachnophyllum esters impacted larvicidal activity against *Aedes aegypti*, *Ae. albopictus*, and *Culex quinquefasciatus*, insecticidal and nematicidal activities against *Callosobruchus maculatus* and *Meloidogyne incognita*, and larvicidal and repellent activity against adults and larvae of yellow fever mosquitos, *Aedes aegypti* (Supplementary Material, Figure S1).

In case of *E. canadensis* EOs, matricaria and lachnophyllum esters were responsible for the antitumoral potential against neoplastic cells K562 and NCI-ADR/RES, fungicidal effects against many fungal strains (*Aspergillus*, *Candida*, *Cryptococcus*, *Rhodotorula*, *Trichophyton*, etc.), and antimicrobial activity against *E. coli* and *C. albican.* Insecticidal potential against larvae and pupae of *Ae. albopictus* and *C. quinquefasciatus* related to the *epi*-bicyclophellandrene present in the oils (Supplementary Material, Figure S2).

## 5. Conclusions

This article provides a comprehensive overview of the subject matter. This review evaluated interspecies and intraspecies compositional variability of the essential oils (EOs) of fourteen *Erigeron* species (native, naturalized, or invasive), such as *E. acris*, *E. annuus*, *E. bonariensis*, *E. breviscapus*, *E. canadensis*, *E. floribundus*, *E. graveolens*, *E. incanus*, *E. mucronatus*, *E. multiradiatus*, *E. philadelphicus*, *E. strigosus*, *E. speciosus*, and *E. sublyratus*. Chemical variability (both quantitative and qualitative) has been demonstrated between different plant organs and at various phenological stages of *Erigeron* sp. As the most common characteristic compounds, limonene, *δ*-3-carene, matricaria ester, lachnophyllum ester, germacrene D, *β*-caryophyllene, *β*-farnesene, *α*-bergamotene, *allo*-aromadendrene, etc., were determined in the oils of the *E. acris*, *E. annuus*, *E. bonariensis*, *E. canadensis*, *E. floribundus*, *E. mucronatus*, and *E. speciosus* plants. Specific constituents, such as monoterpenoid, dendrolasin, diterpenene, neophytadiene, and manool (a labdane type diterpenoid), 2,6,7,7a-tetrahydro-1,5-dimethyl-1H-indene-3-carboxaldehyde, and sesquiterpenoid ledene oxide, were identified in the EOs of *E. bonariensis* L. Appreciable quantities of the rare constituents, 2,3-dimethyl-4(3H)-quinazolinone, and lachnophyllumlactone were identified in the oils of *E. canadensis* and *E. sumatrensis*, respectively. Other prevalent characteristic constituents, such as borneol, bornyl acetate, modhephen-8-*β*-ol, *cis*-arteannuic alcohol, neryl isovalerate, thymol isobutyrate, and *τ*-cadinol, were determined in the *E. graveolens* (*Inula graveolens*) oils. A paucity of data concerning *E. incanus* EOs was revealed, and 3-hydroxy-4-methoxy cinammic acid and thymol acetate were found to be the major ones. The EOs from *E. multiradiatus* and *E. sublyratus* were characterized by matricaria and lachnophyllum esters. The available data on EOs of *E. ramosus* is limited, and the main constituents are known to be *α*-humulene, 1,8-cineole, eugenol, and globulol.

Moreover, the presence of certain significant characteristic compounds in the oils has the potential to function as chemomarkers for the *Erigeron* taxonomic classification.

The different classes of compounds present in the EOs are responsible for various pharmacological and toxic activities, such as anti-inflammatory, anticancer, antiproliferative, skin regeneration, antioxidant, antifungal, antibacterial, insecticidal, larvicidal, repellent, and allelopathic properties, as summarized in this review. The EOs containing appreciable amounts of matricaria and lachnophyllum esters exhibited strong anticancer, anti-inflammatory, antimicrobial, larvicidal, and repellent activities. Repellence also related to borneol, bornyl acetate, caryophyllene derivatives, *τ*-cadinol, modhephen-8-*β*-ol, and *cis*-arteannuic alcohol. Cytotoxicity was determined due to the presence of limonene, *δ*-3-carene, *α*- and *β*-farnesene, (*E*)-*β*-ocimene, ledene oxide, sesquiphellandrene, and dendrolasin in the fleabanes EOs. Skin regeneration and antifungal properties were related to germacrene D, and anti-inflammatory effects were determined due to high amounts of limonene, (*E*)-*β*-ocimene, lachnophyllum ester, and germacrene D. The antimicrobial properties of the oils were conditioned by appreciable quantities of limonene, *β*-pinene, 1,8-cineole, carvacrol, thymol acetae, *β*-eudesmol, 2,6,7,7*α*-tetrahydro-1,5-dimethyl-1H-indene-3-carboxaldehyde, caryophyllene and its oxide, *allo*-aromadendrene, *α*-humulene, farnesene, carvacrol, and eugenol.

This review establishes a foundation for subsequent studies on the secondary metabolites (both volatile and non-volatile) and studies that explore potential sources of new biologically active compounds from other *Erigeron* species. A further review article could be focused on the phytochemistry and biological properties of the plant extracts or individual components from the *Erigeron* genus.

## Figures and Tables

**Table 1:** Data on *Erigeron acris* (L.) EOs published during the period 2000–2025.

Origin of Plant Material	Method of EO Preparation *, Yield	Main Constituents in EOs, %	Literature
Herb material was collected near Białystok, Poland.	HD for 3 h in a Deryng apparatus; 0.3% (per weight of dried plant material).	Limonene 38.8; *β*-pinene + sabinene 15.6; (*E*)-*β*-ocimene 13.5; (*E*,*Z*)-matricaria ester + *α*-murolene 6.0.	[28]
Roots collected in the vicinity of Białystok, Poland.	HD for 3 h of dried material, using a glass Clevenger-type apparatus, 1.0%.	(*Z*,*Z*)-Matricaria ester 49.4; (*Z*)-lachnophyllum ester 37.2.	[29]

* HD—hydro-distillation procedure.

**Table 2:** Main data on *Erigeron annuus* (L.) EOs published during the period 2000–2025.

Origin of Plant Material	Method of EO Preparation *, Yield	Main Constituents in EOs, %	Literature
Aerial parts collected at four different ontogenesis phases: before bud formation, budding, beginning of flowering, full flowering from natural population in Poland.	HD in a glass apparatus for 3 h; 0.08% in fresh herb, 0.13–0.20% in air-dried material.	Germacrene D 42.7–63.7; *β*-caryophyllene 9.0–15.9; (*Z*)-lachnophyllum ester 6.2–8.3.	[32]
Plants harvested at the flowering stage from natural habitat in Poland.	HD of various air-dried plant parts in a glass apparatus for 3 h, 0.47% in flowers and 0.26% in leaves.	Flowers: germacrene D 47.2; (*Z*)-lachnophyllum ester 10.2; matricaria ester 9.5. Leaves: germacrene D 55.6; *β*-caryophyllene 9.4. Stems: germacrene D 44.5; *β*-caryophyllene 13.4; caryophyllene oxide 5.3. Roots: matricaria ester 59.9; (*Z*)-lachnophyllum ester 34.9.	[33]
Roots harvested at the plant full bloom period in the vicinity of Białystok, Poland.	HD of dried plant material by Clevenger-type apparatus for 3h, 0.05%	(*Z*,*Z*)-matricaria ester 45.9; (*Z*)-lachnophyllum ester 27.5; (*Z*,*Z*)-matricaria lactone 7.2.	[29]
Plants at flowering stage collected from sub-alpine Himalayan region of Uttarakhand (7000–10,000 ft elevation), India.	Steam distillation of fresh aerial parts. The distillate was extracted with n-hexane and organic phase separated was extracted once more time with dichloromethane. Both extracts were combined, 0.10–0.20% *v*/*w*.	*cis*-Lachnophyllum ester 68.1; germacrene-D 10.4.	[34]
Plants were collected from cultivated farm at the Department of Cosmetic Science, Hoseo University, Asan, Korea.	Flower blossoms were harvested and air-dried for 24 h. Steam distillation of semi-dried material. From 18 kg of semi-dried flowers was obtained 15 mL of EO.	Germacrene-D 71.0; *trans*-caryophyllene 6.3.	[35]

* HD—hydro-distillation procedure.

**Table 3:** Main compositional data of EOs of *Erigeron bonariensis* L. from various geographical origins.

Origin of Plant Material	Method of EO Preparation *, Yield	Major Constituents in EOs, %	Literature
Collected in five places in the Amazon region (Mato Grosso, Pará and Amapá states).	HD of air-dried (for 7 days) herbs in a Clevenger-type apparatus for 4 h, 0.1–0.5%.	Limonene ≤ 58.4; (*E*)-*β*-farnesene ≤ 30.9; germacrene D ≤ 15.3; (*E*)-caryophyllene ≤ 14.4; *trans*-*α*-bergamotene ≤ 8.1; bicyclogermacrene ≤ 8.3; spathulenol ≤7.6.	[46]
Aerial parts collected in Rio de Janeiro, Brazil.	Distillation of fresh aerial by a modified Clevenger-type apparatus for 8 h, 0.16%.	Limonene 45.0; (*E*)-*β*-ocimene 13.0; (*E*)-*β*-farnesene 6.6; germacrene D 6.4.	[13]
Seeds were cultivated in a greenhouse. Different plat organs (inflorescences (F), leaves (L), stems (S) and roots (R)) collected in Minas Gerais State, Brazil.	Steam distillation of different plant parts in a Clevenger-type apparatus for 90 min; 0.04–0.32%.	F: carvone 21.1. L: Limonene 29.6; *trans*-*α*-bergamotene 10.3; matricaria methyl ester 8.3; *β*-copaen-4*α*-ol 7.4. S: manool 25.3. R: matricaria methyl ester 74.4.	[47]
Aerial parts (at the flowering stage) gathered from Athens, Greece.	HD, 0.30%.	(*Z*)-Lachnophyllum ester 10.8–21.2; (*E*)-*β*-ocimene 11.5–18.9; matricaria ester 9.4–17.6; limonene 8.3–15.1; (*E*)-*β*-farnesene 8.1.	[48]
Aerial parts collected from urban and suburban areas of Southwestern Misiones Province, Argentina	HD of fresh aerial for 2 h in a Clevenger-type apparatus.	Germacrene D 14.6; limonene 13.5; (*E*)-*β*-ocimene 13.3, bicyclogermacrene 6.6; *p*-mentha-1,3,8-triene 5.2.	[49]
Aerial and underground parts at the flowering stage collected in spring, summer and autumn in Monastir, Tunisia.	HD of fresh material for 4 h, using a Clevenger-type apparatus.; yields ranged from 0.02% ± 0.1% to 1.23% ± 0.2% (*w*/*v*, based on fresh plant material).	Spring samples: matricaria ester ≤ 67.3; (*Z*)-nerolidol ≤ 19.9; caryophyllene oxide ≤ 14.3. Summer: matricaria ester ≤ 76.4; (*E*)-*β*-farnesene ≤ 22.7; caryophyllene oxide ≤ 22.6. Autumn samples: matricaria ester ≤ 63.5; geranyl acetone ≤ 25.3; *trans*-*α*-bergamotene ≤ 24.3; limonene ≤ 15.3.	[50]
Aerial parts collected from wastelands in Cagliari, Sardinia, Italy.	HD of dried plant material for 4 h in circularly Clevenger-type apparatus; 0.15%.	*cis*-Lanchnophyllum ester 14.2; (*E*)-*β*-farnesene 12.0, spathulenol 11.4; caryophyllene oxide 10.0; *γ*-muurolene 9.7; limonene 6.5.	[51]
Aerial parts collected from two different localities: from roadsides and more or less anthropized wastelands in Monastir region, Tunisia.	HD of dried plant material for 4 h in a Clevenger-type apparatus; 0.19%. Supercritical CO_2_ extraction (SFE) performed in a laboratory apparatus; 1.9 × 10^−2^%.	By HD: caryophyllene oxide 18.7; spathulenol 18.6; *α*-curcumene 10.2; carvacrol 9.8; neophytadiene 6.1; limonene 5.1. By SFE: neophytadiene 53.2; carvacrol 12.9; (*E*)-*β*-farnesene 7.3; spathulenol 7.2; *α*-curcumene 6.8.	[51]
Leaves collected in Sector Chama, Mérida State, Venezuela.	HD of fresh leaves for 4 h using a Clevenger-type apparatus; 0.04% (*v*/*w*) (on a dry weight of the plant material).	(*E*)-*β*-Farnesene 37.8; *trans*-*β*-ocimene 20.7; *β*-sesquiphellandrene 9.8; *α*-farnesene 5.6; limonene 5.1; (*Z*)-*β*-ocimene 5.1.	[52]
Plants collected from forests and adjoining areas of Gorakhpur Division (about 91m above sea level) in Eastern Uttar Pradesh, India.	HD of twigs using a Clevenger apparatus (for 4 h at 90 ± 2 °C); 0.06% (on fresh weight basis).	Not indicated.	[53]
Material (under name *C. linifolia)* collected during flowering stage from Nubaria, Alexandria, Egypt.	HD of dried powdered aerial parts by a Clevenger-type apparatus at 50 °C for 3 h.	Bergamotene 27.4; D-limonene 22.6; carvone 5.9; *β*-farnesene 5.7.	[54]
Plant material was collected from Dineshpur, Udham Singh Nagar district, India.	SD of fresh aerial parts, then distillates were extracted with n-hexane and dichloromethane; 0.75% (*v*/*w*).	*β*-Eudesmol 40.6; caryophyllene oxide 34.1; carvacrol 8.9.	[55]
Leaves gathered in Kabianga location, Kericho, Kenya.	HD of fresh leaves for 4 h using a Clevenger-type apparatus; 0.04% (on dry weight).	2,6,7,7a-Tetrahydro-1,5-dimethyl-1H-indene-3-carboxaldehyde 49.1; limonene 8.3; *β*-pinene 5.4.	[56]
Aerial parts collected in the Atlantic Forest, in Parana State, Brazil.	HD for 4 h and 30 min in a Clevenger-type apparatus, using fresh or dried plant material; 1.23% (fresh samples) and 0.86% (dried material).	Limonene 66.3; 2-heptyl acetate 6.9.	[57]
Aerial parts collected from Bach Ma National Park, Thue Thien, Hue province, Vietnam.	HD of fresh aerial parts (leaves, stems, and flowers) for 4 h using a Clevenger-type apparatus; 1.10%.	*allo*-Aromadendrene 41.2; *β*-caryophyllene 13.3; caryophyllene oxide 12.2; *α*-humulene 5.4.	[58]
Plants collected from two populations along the Cairo–Alexandria desert road, Egypt.	HD of air-dried powder of the above-ground parts in a Clevenger-type apparatus for 3 h; 0.049%.	*trans*-*α*-Farnesene 25.0; *o*-ocimene 12.6; ledene oxide 10.9; dendrolasin 8.4; *α*-maaliene 6.6.	[59]
Branches and leaves of *Conyza bonariensis* (L.) collected from the Medicinal Plant Garden, Federal University of Paraíba (UFPB), João Pessoa, Paraíba, Brazil.	HD in a Clevenger-type apparatus.	Sesquicineole 48.5; sesquisabinene 10.9; limonene 9.6; thymol 6.2.	[60]
Whole plants collected in Dagni Koudzragan, Togo.	SD using a Clevenger-type apparatus for to 4 h; 0.12% of fresh material; 0.3% from flowers, 0.01% from roots.	Whole plant: methyl *cis*-lachnophyllum ester 58.0; limonene 11.7; *β*-ocimene 8.3, *β*-farnesene 6.2. Leaves: *β*-caryophyllene 16.2, *β*-farnesene 15.5; limonene 12.8, methyl *cis*-lachnophyllum ester 9.8; germacrene-D 6.8; *β*-ocimene 5.8; *γ*-cadinene 5.3. Flowers: *β*-caryophyllene 21.1; *β*-farnesene 12.9; *γ*-cadinene 5.7; *allo*-aromadendrene 5.7; limonene 5.6. Roots: methyl *cis*-lachnophyllum ester 50.6; *β*-farnesene 25.7.	[39]
Plants from the Medicinal Plant Garden, UFPB, João Pessoa, Paraíba, Brazil.	HD of branches and leaves in a Clevenger-type apparatus for 2 h; 1.3% (*w*/*w*).	(*Z*)-2-Lachnophyllum ester 57.2; limonene 14.3.	[61]
Leaves and stems collected from the Bio-Park at Bahauddin Zakaria University, Multan; and from a hilly area of Abbottabad, Pakistan.	SD for 4 h of fresh parts on the same day of their gathering. The distillate was extracted in n-hexane; 0.21% (*w*/*w*, on fresh plant material).	Matricaria ester 43.1; *cis*-lachnophyllum ester 24.9; *trans*-*β*-farnesene 10.2.	[62]

* HD—hydro-distillation procedure; SD—steam distillation procedure.

**Table 4:** Main compositional data of the EOs of *Erigeron canadensis* L. (*Conyza canadensis* (L.) Cronq.).

Origin of Plant Material	Method of EO Preparation *, Yield	Major Constituents in EOs, %	Literature
Wild-growing plants collected at the flowering stage near Lodz, Poland.	HD of air-dried material in a glass apparatus for 5 h, 0.8%	Limonene 70.0; *trans*-*α*-bergamotene 7.0.	[67]
From different parts (herb, leaves, flowers, stems, roots) at various ontogenesis phases; plants collected in Łódź, Poland; Alps, France; Rome, Italy; Seville, Spain; Belgium; Plovdiv, Bulgaria; Vilnius, Lithuania and Israel.	HD; EO yield of herb was the highest at early flowering phases; ≤0.8%.	R-(+)-limonene 51.4–87.9, *trans*-*α*-bergamotene 5.4–11.9; germacrene D 7.3; (*Z*,*Z*)-matricaria ester ≤7.7; (Z)-*β*-farnesene ≤6.3; (*E*)-*β*-ocimene 5.1–13.4.	[68]
Plant material collected in France.		Limonene 76.03 ± 0.07; *α*-santalene 5.84 ± 0.04.	[69]
Harvested at six different phases of vegetation (from before budding until the phase of full deflorate) near Plovdiv, Bulgaria.	HD in a modified laboratory glass apparatus for 2 h The oil content increased until the budding phase (0.25%), and remained the same until the phase of blossom fall with seed development and then, in the phase of full defloration, it reduced by half.	Limonene 77.7–89.4, *β*-pinene ≤ 6.6.	[70]
Aerial parts harvested at flowering stage around Kerman, Kerman Province, Iran.	HD using a Clevenger-type apparatus for 3 h, 0.5% (*w*/*w*).	(*E*)-*β*-Farnesene 14.6; spathulenol 14.1; limonene 12.3; myrcene 8.9; *ar*-curcumene 7.8; *iso*-spathulenol 7.7; phytol 7.3; zingiberene 5.5.	[71]
Plant material collected at vegetative, flowering and flowering–fruiting stages in Athens, Greece.	HD using a Clevenger-type apparatus.	Limonene 50.0–70.3; *β*-pinene ≤9.5; matricaria ester ≤14.4; (*E*)-*β*-ocimene ≤7.5.	[48]
Plant material collected in Korea.	SD.	D, L-limonene 68.3; *δ*-3-carene 15.9.	[72]
	Fresh aerial parts by HD and SD.	Obtained by HD: limonene 68.9.	[73]
	SD	Limonene, *α*-bergamotene, (*E*)-*β*-farnesene, (*Z*)-*β*-farnesene, 2,3-dimethyl-4(3H)-quinazolinone.	[74]
Herb and roots collected in Szeged and Jakabszállás, Hungary.	HD of dried flowering shoots and roots for 2 h; from herbs 0.72%, in roots 0.20% (on the basis of the dry weight).	Aerial parts: limonene 79.2. Roots: 2*Z*,8*Z*-matricaria ester ≤93.9.	[75]
Commercial EO purchased from Essential Oil University, Charlestown, IN, USA.		Limonene 48.2.	[76]
Aerial parts were collected from Mekelle, Ethiopia.	HD of dried aerial parts in a Clevenger apparatus for 3 h; 0.69% (w/fresh weight).	Limonene 57.2; caryophyllene 6.7.	[77]
Plants were collected in China.		Limonene 14.8; *epi*-bicyclosesquiphellandrene 11.0.	[78]
Plants in Gorakhpur Division, Eastern Uttar Pradesh, India.	HD of twigs using Clevenger’s apparatus for 4 h at 90 ± 2 C; 0.1% on a fresh weight basis.	Not indicated.	[53]
The aerial parts (during the flowering period) and roots were collected from Manavgat, Antalya, Turkey.	HD of fresh aerial parts and roots for 3 h, using a Clevenger-type apparatus; 0.17% in aerial parts and traces in roots.	Aerial parts: limonene 28.1; spathulenol 16.3; *β*-pinene 9.7. Roots: *cis*-lachnophyllum ester 86.5.	[79]
Aerial parts of *C. canadensis* growing wild in Kashmir valley, India.	HD	Limonene 23.8; (*Z*)-lachnophyllum ester 21.3; (*E*)-*β*-ocimene 16.0; *β*-pinene 11.8; (*E*)-*β*-farnesene 7.8.	[80]
The plants were collected (leaves and roots) in two hours of the day, 6 a.m. and 4 p.m.; gathering was carried out in a rural area in the municipality of Naviraí, Mato Grosso do Sul, in the center-west region of Brazil.	HD in a Clevenger modified apparatus for 4 h; EOs yields varied in leaves from 1.2 ± 0.3 to 0.7 ± 0.2; in roots from 0.6±0.2 to 0.4 ± 0.1 during collection at 6 a. m. and 4 p.m.	Leaves: limonene 61.0 and 38.0; caryophyllene oxide 12.5 and 22.3; spathulenol 5.4 and 10.7 at 6 a.m. and 4 p.m., respectively. Roots: lachnophyllum methyl ester 93.7 and 91.6; matricaria methyl ester 5.2 and 6.7, when the plants collected 6 a. m. and 4 p.m., respectively.	[81]
Fresh aerial parts of wild grown plants collected from the suburbs of District Abbottabad, Pakistan.	SD of fresh aerial parts in a stainless-steel distiller. The distillate was collected in a separating funnel for 3 h, 0.22%.	Limonene 41.3; germacrene D 10.3; matricaria ester 10,3; *trans*-*β*-ocimene 8.2; *cis*-lachnophyllum ester 6,5; (3*E*,5*E*)-2,6-dimethyl-1,3,5,7-octatetraene 6.9.	[82]
Aerial parts (leaves, stems, and flowers) collected from Bach Ma National Park, Thue Thien Hue province, Vietnam.	HD of fresh aerial parts for 4 h, using a Clevenger-type apparatus; 1.37%.	Limonene 41.5; *β*-pinene 8.8; (*Z*)-lachnophyllum ester 5.5.	[58]
Leaves and stems collected from the Bio-Park at Bahauddin Zakaria University, Multan; and from a hilly area of Abbottabad, Pakistan.	SD for 4 h of fresh parts of plants on the same day as their collection. The distillate was extracted in n-hexane, 0.21% (*w*/*w* on fresh plant material).	Matricaria ester 31.7; limonene 28.4; *cis*-lachnophyllum ester 16.3; germacrene D 6.4; *trans*-*β*-ocimene 5.0.	[62]
The aerial parts of wild plants collected from the Al Mansour neighborhood, Al-Jubeiha, Amman governorate, Jordan.	HD of fresh organs (inflorescence heads (Inf), leaves (L) and stems (St)) for 3 h in a Clevenger-type apparatus. Inf: 0.38%, L: 1.71%, St: 0.023%.	(2*E*,8*Z*)-Matricaria ester: 60.7 (L), 31.6 (St), 15.4 (Inf).	[83]

* HD—hydro-distillation procedure; SD—steam distillation procedure.

**Table 5:** Main compositional data of EOs of *Erigeron floribundus* (H.B. et K.) Sch. Bip. (*Conyza floribunda* H.B. et K.).

Origin of Plant Material	Method of EO Preparation *, Yield	Main Constituents in EOs, %	Literature
Young plants (identified as *Conyza albida* Willd. ex Sprengel) collected from a natural population in the University of Athens Campus, Greece.	HD of fresh aerial parts (rosette) for 3 h, using a modified Clevenger-type apparatus; 0.25%.	*cis*-Lachnophyllum ester 30.0; germacrene D 12.9; (*E*)-*β*-farnesene 12.5; limonene 11.2; (*E*)-*β*-ocimene 8.4; lachnophyllumlactone 7.1	[86]
Plant material (identified as *C. albida*) from Greece.	HD.	*cis*-Lachnophyllum ester 8.8–36.5; germacrene D 10.5–20.2; limonene 10.0–21.1.	[48]
*C. sumatrensis* (Retz.) E. Walker var. *sumatrensis* and C. *sumatrensis* (Retz.) E. Walker var. *floribunda* (Kunth) J. B. Marshall collected from urban and suburban areas of Southwestern Misiones Province, Argentina.	HD of fresh aerial parts for 2 h. in a Clevenger-type apparatus.	Limonene ≤ 63.1; (*E*)-*β*-ocimene ≤ 8.7; germacrene D 7.5; *β*-pinene ≤ 6.3; bicyclogermacrene 5.3.	[49]
Leaves and flowers of the plants (identified as *Erigeron floribundus* (H.B. et K.) Sch. Bip.) collected in Yaoundé (Centre province), and Bafoussam and Dschang (Western province), Cameroon.	HD for 3 h, using a Clevenger-type apparatus; 0.2% from leaves and 0.3% from flowers.	Fower EO: (*E*)-*β*-farnesene 22.3–24.1; *β*-caryophyllene 17.3–20.1; germacrene D 10.1–11.0. Leaf EO: (*Z*)-2-lachnophyllum ester 23.7–26.2; (*E*)-*β*-farnesene 14.6–16.4; *β*-caryophyllene 14.7–16.6; limonene 9.5–11.4.	[87]
leaves, flowers and root bark of the plants (identified as *Conyza sumatrensis* Retz E. K. Walker) collected in Côte d’Ivoire.	HD.	Leaf and flower EOs: limonene 13.0–25.5; (*E*)-*β*-farnesene 7.8–17.5; (*E*)-*β*-caryophyllene 9.1–15.8; germacrene D ≤13.6. Root bark oil: (*Z*)-lachnophyllum acid methyl ester 75.0.	[88]
The aerial parts of *E. floribundus* collected from the campus of Regional Medical Research Centre (RMRC), (ICMR), Belgaum (at a height of 800 m.), Karnataka, India.	SD using copper still fitted with spiral glass condensers for 3 h, then extracted with n-hexane and dichloromethane, 0.01% (*v*/*w*).	Not presented	[89]
*Conyza sumatrensis* (Retz.) E. Walker collected at the flowering stage in the area of Monastir, Tunisia.	HD of different parts (flower heads, leaves, stems, and roots).	Leaf EO: caryophyllene oxide 20.5; spathulenol 13.8; matricaria ester 7.5. Root EO: matricaria ester 74.3.	[90]
Aerial parts harvested in Dschang, West Province of Cameroon (1450 m a.s.l.).	HD of dry aerial parts in a Clevenger-type apparatus for 4 h, 0.2% (*n* = 3) (*w*/*w*), on a dry-weight basis.	Caryophyllene oxide 12.4; spathulenol 12.2; limonene 8.8; (*E*)-*β*-farnesene 5.5.	[91]
*Conyza sumatrensis* (Retz.) collected from two different areas of Abbottabad, Pakistan.	SD of fresh aerial parts in a stainless-steel distillation apparatus for 4 h; 0.25%.	*cis*-Lachnophyllum ester 37.7; limonene 21.6; germacreneD 13.4; *trans*-*β*-farnesene 6.6; *trans*-*β*-ocimene 5.7; elixene 5.3.	[92]

* HD—hydro-distillation procedure; SD—steam distillation procedure.

**Table 6:** Compositional data of EOs of *Inula graveolens* published after 2022.

Origin of Plant Material	Main Constituents in EOs, %	Literature
Aerial parts of *Inula graveolens.* EOs obtained by HD.	EO from aerial parts: borneol 28.8; caryophylla-4(14),8(15)-dien-6-ol 11.5; caryophyllene oxide 10.9; *τ*-cadinol 10.5; bornyl acetate 9.4. Root EO: modhephen-8-β-ol 24.7; *cis*-arteannuic alcohol 14.8; neryl isovalerate 10.6; thymol isobutyrate 8.5.	[95]

**Table 7:** Compositional data of EOs of *Erigeron incanus* Vahl L. (published 2000–2025).

Origin of Plant Material	Method of EO Preparation *, Yield	Main Constituents in EOs, %	Literature
The aerial parts were collected from Sana’a city, Yemen.	HD of fresh aerial parts for 4 h, using a Clevenger-type apparatus, 1.8%, (*w*/*w*) (on dry weight).	3-hydroxy-4-methoxy Cinammic acid 72.6; thymol acetate 11.3.	[24]

* HD—hydro-distillation.

**Table 8:** Compositional data of EOs of *Erigeron mucronatus* DC published during the period 2000–2025.

Origin of Plant Material	Method of EO Preparation *, Yield	Main Constituents in EOs, %	Literature
Stems, leaves, flowers and roots of the plants, growing wild in rocky areas of the Nilgiri Hills, India.	HD of leaves in a Clevenger apparatus for 2 h; 0.5%. The yield varied from 0.49 to 0.58%, being highest during the rainy season.	(*E*,*E*)-Matricaria ester 13.7; caryophyllenes 11.4; limonene 10.3; *cis*-methyl lachnophyllum ester 8.7; germacrene-D 6.3.	[25]
Plants (identified as *E. mucronatus* DC. *E. karwinskianus* DC.) collected from sub-alpine Himalayan region of Uttarakhand (7000–10,000 ft elevation) at flowering stage.	SD, extraction in n-hexane and dichloromethane, 0.1% (*v*/*w*) in *E. mucronatus* and 0.2% (*v*/*w*) for *E. karwinskianus.*	*E. karwinskianus* DC: *trans*-2-*cis*-8-Matricaria ester 25.4; germacrene-D 13.1; *α*-muurolene 6.4; *β*-(*E*)-ocimene 6.0; *β*-elemene 6.0. *E. mucronatus: trans*-2-*cis*-8-matricaria ester 62.1; *cis*-lachnophyllum ester 21.2.	[34]

* HD—hydro-distillation procedure; SD—steam distillation procedure.

**Table 9:** Compositional data of EOs of *Erigeron ramosus* published during the period 2000–2025.

Origin of Plant Material	Main Constituents in EOs, %	Literature
Korea (*E. ramosus* is an alien species in Korea)	*β*-Caryophyllene 24.0; *α*-humulene 14.5; 1,8-cineole 9.0; eugenol 7.2; globulol 7.1; caryophyllene oxide 5.2.	[103]

**Table 10:** Compositional data of EOs of *Erigeron sublyratus* (published in 2000–2025).

Main Constituents in EOs, %	Literature
Lachnophyllum ester 53.4–64.2; germacrene D 5.6–8.6; *trans*-*β*-ocimene ≤ 7.5; *β*-caryophyllene ≤ 6.8; *β*-myrcene ≤ 6.3 ; (*E*)-*β*-farnesene ≤ 5.0 .	[105]

## Data Availability

Data are contained within the article and Supplementary Materials.

## Supplementary Materials

The following supporting information can be downloaded at: https://www.mdpi.com/article/10.3390/molecules30142989/s1, Figure S1: Principal component biplot of PC1 and PC2 scores demonstrates the relationships between major components of *E. bonariensis* EOs and various biological activities; Figure S2: Principal Component Analysis (PCA) of *Erigeron canadensis* EOs, biplot illustrates the relationships between major components of the oils and various biological activities.
