# Supplementary material for: Compositional Variability of Essential Oils and Their Bioactivity in Native and Invasive Erigeron Species
Source: Molecules. 2025 Jul 16;30(14):2989. doi: 10.3390/molecules30142989 (PMC12298210; doi:10.3390/molecules30142989)
Supplement: Supplementary file 1 [file molecules-30-02989-s001.zip › molecules-3738780-supplementary.pdf]

Review

Compositional Variability of Essential Oils and Their Bioactivity in Native and Invasive *Erigeron* Species

Asta Judžentienė [1,2]

[1] Life Sciences Center, Institute of Biosciences, Vilnius University, Saulėtekio Avenue 7, LT-10257 Vilnius, Lithuania; asta.judzentiene@gmc.vu.lt or asta.judzentiene@ftmc.lt
[2] Center for Physical Sciences and Technology, Department of Organic Chemistry, Saulėtekio Avenue 3, 10257 Vilnius, Lithuania

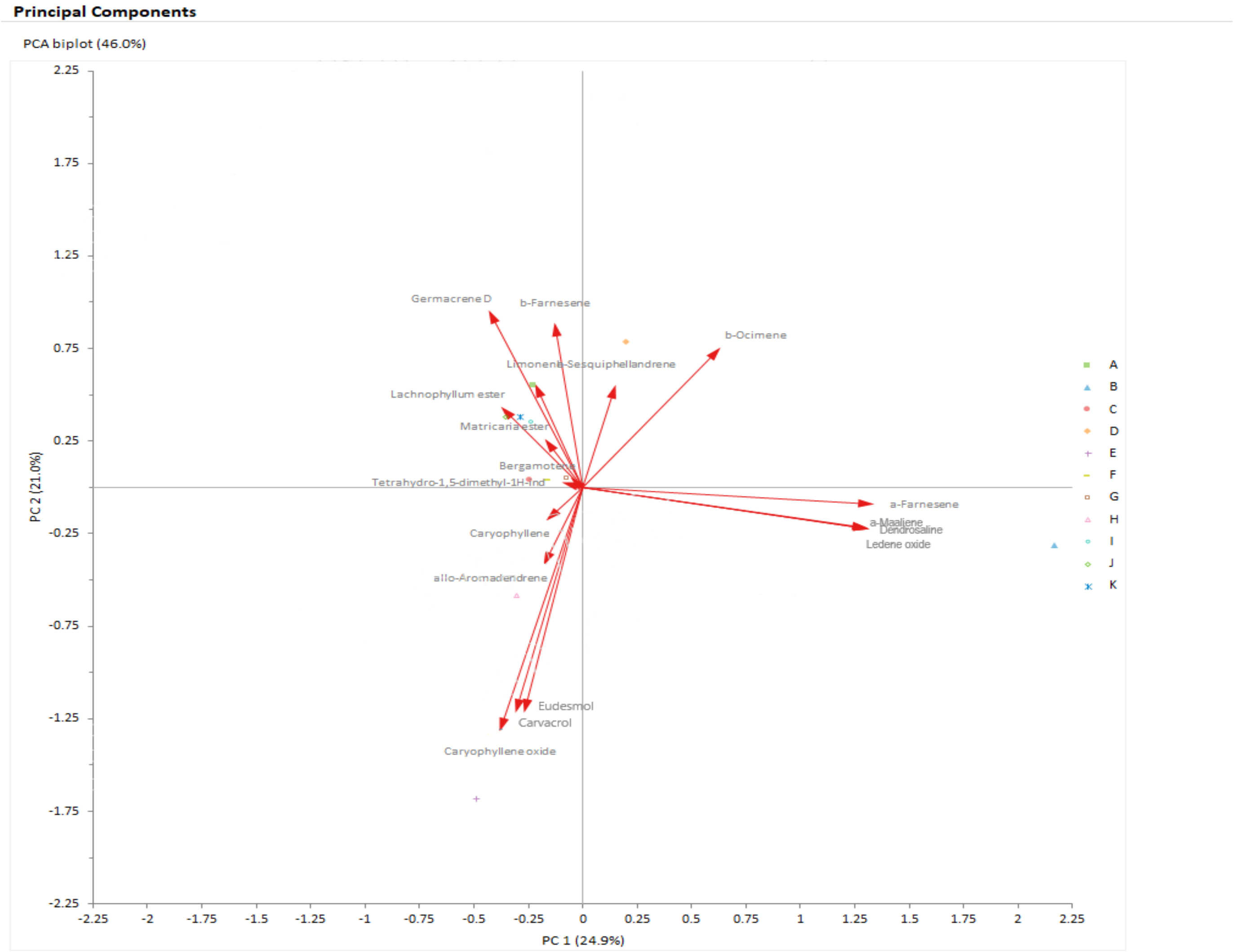

Figure S1. Principal component biplot of PC1 and PC2 scores demonstrates the relationships between major components of *E. bonariensis* EOs and various biological activities: A–anti-inflammatory activity (inhibition of the LPS-induced inflammation) [13]; B–cytotoxicity against HepG2, and inhibitory effects of

the collagenase, elastase, hyaluronidase and tyrosinase [59]; C–anticancer activity human tumor cell lines and non-tumor keratinocyte lines, toxicity and anti-aging properties [61]; D–cytotoxicity activity against HeLa, A-459 and MCF-7 human cell lines, and against normal Vero cells; antimicrobial effects against *B. cereus, S. epidermidis* and *C. albicans* [52]; E and F and G–antibacterial activity against *S. enterica*, *P. aeruginosa, E. coli* [31], *E. coli, S. typhi* [56]; G–antimicrobial effects against *B. subtilis;* and insecticidal and larvicidal activity against adults and larvae of *C. pipiens* mosquitoes [54]; H–larvicidal activity against *Aedes aegypti*, *Ae. Albopictus* and *Culex quinquefasciatus* [58].; I, J–insecticidal and nematicidal activities against adults of cowpea weevil *Callosobruchus maculatus* and root-knot nematode *Meloidogyne incognita* of EOs from ground parts and roots, respectively [39], K–larvicidal and repellent activity against adults and larvae of yellow fever mosquitos, *Aedes aegypti* [62].

Percentage of the following constituents in *E. bonariensis* EOs: limonene, *β*-ocimene, carvacrol, *α*-farnesene, *β*-farnesene, *allo*-aromadendrene, germacrene D, *α*-maaliene, lachnophyllum ester, matricaria ester, *β*-sesquiphellandrene, bergamotene, caryophyllene, caryophyllene oxide, eudesmol, ledene oxide, dendrolasin and tetrahydro-1,5-dimethyl-1H-indene-3-carboxaldehyde were chosen as variables.

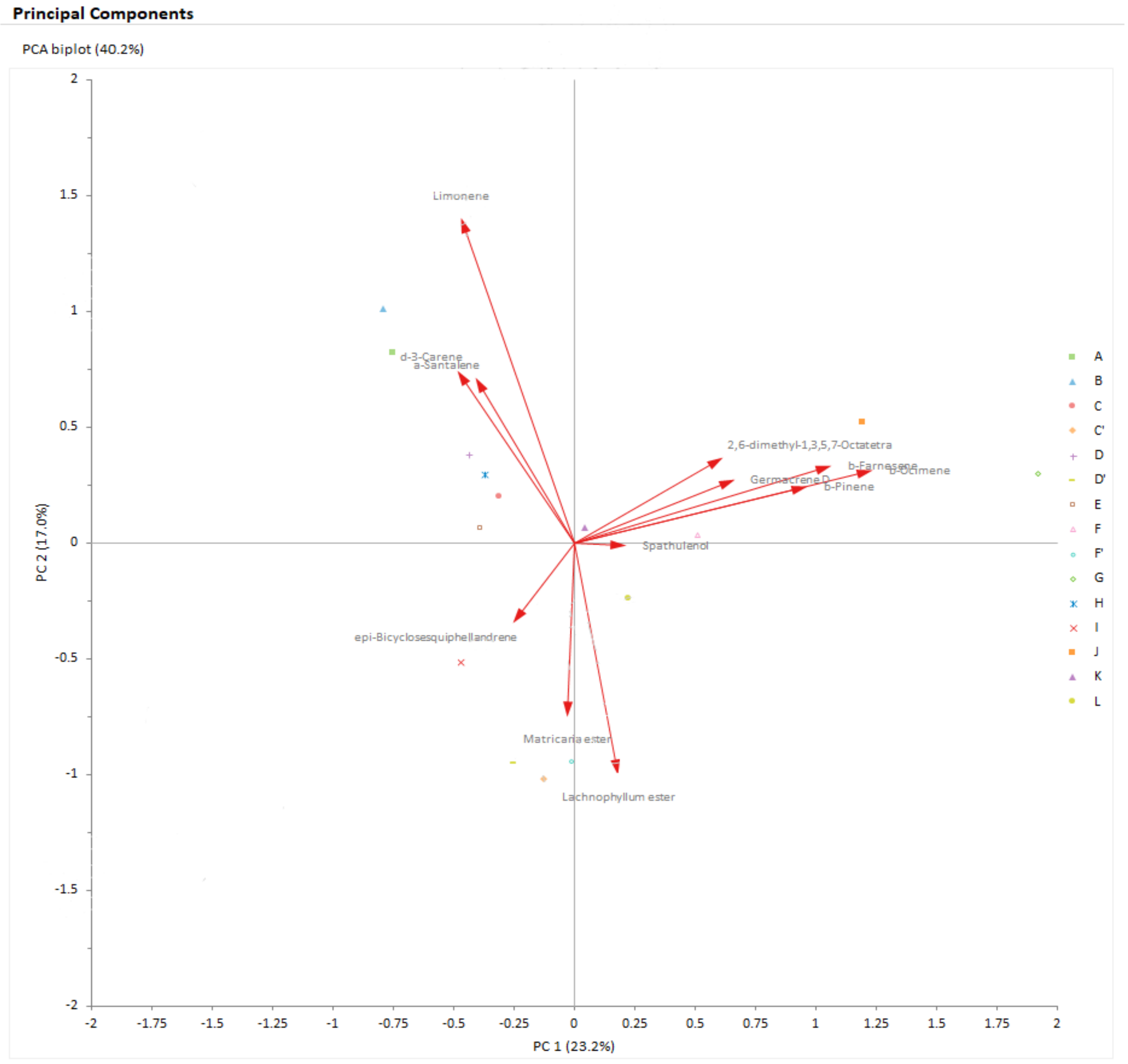

Figure S2. Principal Component Analysis (PCA) of *Erigeron canadensis* EOs, biplot illustrates the relationships between major components of the oils and various biological activities: A–cytotoxic activity using HaCaT keratinocyte cells [72]; B–antifungal effects against *R. solani, F. solani* and *C. lindemuthianum* [69]; C, C′– antitumoral potential against neoplastic cells K562 and NCI-ADR/RES for EOs of leaves and roots, respectively [81]; D, D′–fungicidal effects against many fungal strains (*Aspergillus, Candida, Cryptococcus*, *Rhodotorula*, *Trichophyton*, *etc.*) of EOs of leaves and roots, respectively [75]; E–antimicrobial activity against three bacteria (*S. enteritidis*, *S. aureus*, *P. aeruginosa*) and three fungi (*A. alternata*, *A. niger* and *P. digitatum*) [76]; F, F′–antimicrobial activity against *E. coli* and *C. albicans,* EOs from aerial parts and roots, respectively [79]; G–antibacterial activity against *C. albicans* and *C. parapsilosis*, and antioxidant potential [80]; H–allelopathic properties on seed germination of *Brassica chinensis* Linn., *B. pekinensis* Rupr., *Triticum sestivum* Linn., *Sorghum bicolor* (Linn.) Moench [73,74]; I–insecticidal potential against larvae and pupae of *Aedes albopictus* and *Culex quinquefasciatus* [78]; J–repellence against dengue mosquito, *Aedes aegypti* [82]; K–larvicidal activity against *A. aegypti, A. albopictus* and *C. quinquefasciatus* [34] and L–larvicidal and repellent effects against *A. aegypti* [62].

Percentage of the following constituents in *E. canadensis* EOs: limonene, δ-3-carene, β-pinene, β-ocimene, 2,6-dimethyl-1,3,5,7-octatetraene, α-santalene, β-farnesene, germacrene D, lachnophyllum ester, matricaria ester, *epi*-bicyclosesquiphellandrene and spathulenol were chosen as variables.
